# Role of Morphological Structure, Doping, and Coating of Different Materials in the Sensing Characteristics of Humidity Sensors

**DOI:** 10.3390/s140916343

**Published:** 2014-09-03

**Authors:** Ashis Tripathy, Sumit Pramanik, Jongman Cho, Jayasree Santhosh, Noor Azuan Abu Osman

**Affiliations:** 1 Department of Biomedical Engineering, University of Malaya, Kuala Lumpur 50603, Malaysia; E-Mail: jsanthosh@um.edu.my; 2 Department of Biomedical Engineering, Inje University, Gimhae 621-749, Korea; E-Mail: minerva@inje.ac.kr

**Keywords:** humidity sensor, relative humidity, stability, response time, miniaturization

## Abstract

The humidity sensing characteristics of different sensing materials are important properties in order to monitor different products or events in a wide range of industrial sectors, research and development laboratories as well as daily life. The primary aim of this study is to compare the sensing characteristics, including impedance or resistance, capacitance, hysteresis, recovery and response times, and stability with respect to relative humidity, frequency, and temperature, of different materials. Various materials, including ceramics, semiconductors, and polymers, used for sensing relative humidity have been reviewed. Correlations of the different electrical characteristics of different doped sensor materials as the most unique feature of a material have been noted. The electrical properties of different sensor materials are found to change significantly with the morphological changes, doping concentration of different materials and film thickness of the substrate. Various applications and scopes are pointed out in the review article. We extensively reviewed almost all main kinds of relative humidity sensors and how their electrical characteristics vary with different doping concentrations, film thickness and basic sensing materials. Based on statistical tests, the zinc oxide-based sensing material is best for humidity sensor design since it shows extremely low hysteresis loss, minimum response and recovery times and excellent stability.

## 1. Introduction

Humidity, which is amount of water vapor present in air or atmosphere, is highly variable and changes according to seasons, land, sea, temperature and so on, and has an important role in the quality of industrial products, advanced instruments and human life. The regulation of humidity is important for human comfort, storage of various goods, industrial process control, high-tech instruments, and plenty of advanced sectors [1]. In industry, optimum humidity conditions should be provided on production lines for obtaining high quality products. For example, in textile processing, generation of electrostatic charges during the fabrication may cause the materials to cling. This is prevented by keeping the environment in damp conditions. On the other hand, dry conditions are required during processing of silicon wafers in a clean room, assembling electrical products on the assembly line and so on. In the automobile industry, humidity sensors are used in rear window defoggers and motor assembly lines. In the agriculture sector, since adequate environmental humidity conditions are needed to grow fruits and vegetables, similar conditions are needed in the preservation of different types of foods and cottons. There are many domestic applications, such as cooking control for microwave ovens, intelligent control of laundry and the living environment in buildings, and intelligent control and so on. In the medical field, humidity sensors are used in respiratory equipment, sterilizers, and incubators, pharmaceutical processing, and biological products. Thus, the measurement and control of humidity have significant importance in many areas for different purposes.

**Figure 1.**
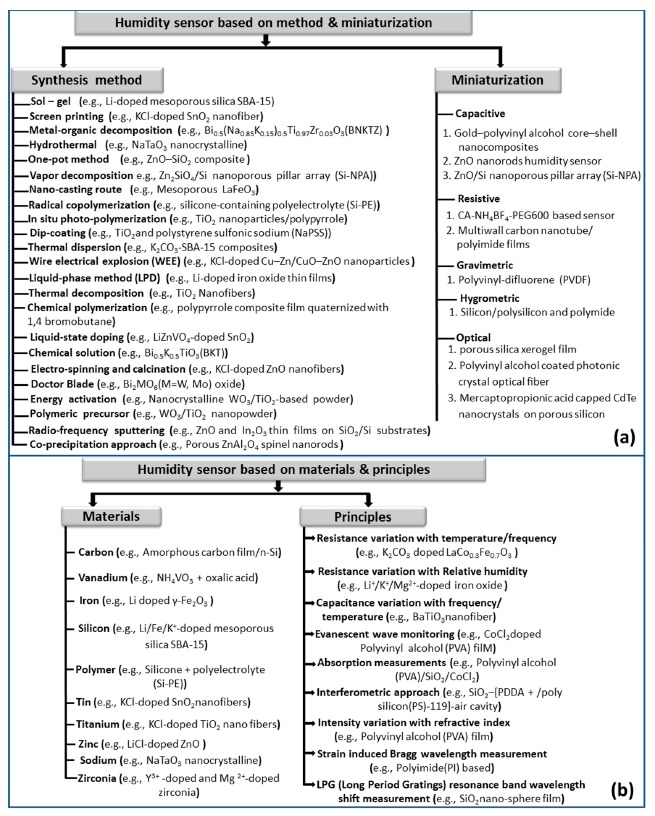
(**a**) Humidity sensors based on synthesis method and miniaturization; (**b**) Humidity sensors based on materials and principle.

In 1450, Nicolas Cryfts invented a hygrometer which is the first humidity measurement instrument on record. This hygrometer used wool to determine the changes of humidity in air [2,3]. Over the past 550 years, many other kinds of hygrometer have been invented. A century later, in 1550, the device was improved by substituting a sponge for the wool and various versions of the hygrometer were devised. Subsequently, the sponge was replaced by paper, hair, nylon, and acetate. During the seventeenth and eighteenth centuries, there were several opinions about how water dissolves in air. It was also established that a relationship exists between humidity and frequency or temperature. Currently, miniaturized humidity sensors have shown many advantages, including integration, small size, low power consumption, high performance, low cost, and ease of mass fabrication, compared to the classical measurement used in different hygrometers such as lithium chloride (LiCl), dew point, and chilled mirror type sensors [4]. The different types of humidity sensors are classified according to the working technology and sensing principle and illustrated in Figure 1a,b.

Humidity is evaluated by different functions such as vapor pressure, saturation vapor pressure, dew/frost point temperature, and relative humidity [5]. Vapor pressure (hPa) is a partial pressure of water vapor in the air. Saturation vapor pressure is measured on a surface of water or ice at thermodynamic equilibrium condition. Dew point is a temperature, above 0 °C, at which the air will be cooled down to reach saturation at constant pressure condition and it is generally equal to or lower than the actual air temperature. Frost point is a temperature, below 0 °C, at which moist air saturates with respect to ice.

Relative humidity (RH, %) is a ratio of the vapor pressure (VP) of moist air to its saturation vapor pressure (SVP) at a given temperature, which is expressed by Equation (1):

(1) $$\text{RH}=\frac{VP}{\text{SVP}}\times 100\%$$

Humidity can also be expressed in other ways like absolute humidity (g/m^3^), defined as the amount of water vapor contained in a unit volume of dry air, *i.e.*, mixing ratio m_r_ (parts per million by volume, ppmv) and the ratio with respect to saturation is defined as saturation deficit p_sd_ (mbar).

Development of an ideal humidity sensor depends on some key criteria, such as accuracy, power consumption, precision, repeatability, long-term stability, response time, size, packaging, and cost. In recent advances, cost effective miniaturization with ultra-high precise performance has received much attention for advanced application fields. To know the effect of different doping elements or materials in the sensor materials is most vital part of humidity sensor development for the different applications.

In this review, we aim to present extensive researches and developments of humidity sensing materials and characteristics for a wide variety of applications. Particularly changes in impedance or resistance, capacitance, hysteresis, recovery and response times, and stability with respect to relative humidity, frequency, and temperature are the primary interest of this present review article. Applications of humidity sensors in each field require different operating conditions, and various types of humidity sensing mechanisms based on a variety of sensing materials will be described. A typical comparative study of the above particular characteristics changes with different materials is critically reviewed in the following sections. Due to heavy population growth and their daily activities, environmental pollution is a great problem for a healthy atmosphere. Hence different types of gas sensors have an important role to detect or analyze the different contaminants present in the atmosphere. Importantly, the detection of humidity is one of the most important tasks owing to its versatile application in the fields of industrial control, agriculture, material analysis and biomedical instruments. A potential versatile humidity sensor must have good reproducibility, low hysteresis, low cost, be resistant against contaminants, good sensitivity, good durability and long life time, a very short response time, and low dependency on temperature.

This review paper is organized as follows: the general notion and definitions of humidity are presented at the beginning of the Introduction section. Section 2 highlights the miniaturization of humidity sensors with their potential advantages and disadvantages. This section mainly discusses the distinct transduction principles of different types of humidity sensors such as resistive, capacitive, hygrometric, gravimetric, and optical sensors. The essential physical or morphological properties and typical electrical characteristics of relative humidity sensors based on different key materials such as carbon, polymer, silicon, vanadium, iron, tin, titanium, zinc, zirconia, and sodium are highlighted in Section 3. This section also focuses the changes in the physical, chemical, electrical as well as structural characteristics of the base material due to different coatings or dopants. Section 4 follows with a brief review where a number of key applications of humidity and moisture measurement are highlighted in areas such as structural health monitoring (SHM), food processing and storage, medicine, ecology, agriculture, mineral processing, fuel quality control, and aerospace. Finally the concluding remarks are provided in Section 5.

## 2. Miniaturized Humidity Sensors

For many sophisticated applications, there has been a keen attempt to reduce the size of the sensors using the most advanced technologies collectively called miniaturization. Miniaturized humidity sensors mainly depend on five different transduction principles: hygrometry, capacity, resistance, gravimetry and optical properties.

### 2.1. Hygrometric Sensors

**Figure 2.**
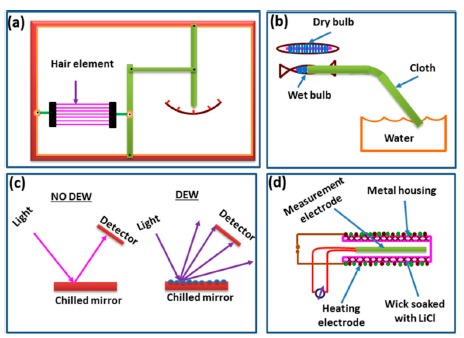
Fundamental schematic representation of different hygrometric sensors. (**a**) mechanical; (**b**) dry bulb-wet bulb; (**c**) chilled mirror; (**d**) LiCl dew point hygrometer.

For many decades hygrometric sensors have been using in old devices that are classified into four basic types—mechanical, dry bulb-wet bulb, chilled mirror, LiCl dew point—for the measurement of the humidity of air, which is converted into mechanical energy [6–10]. These are illustrated in Figure 2a–d.

Generally, hygrometric sensors have two different materials with different thermal expansions coefficients, such as a polysilicon membrane and a hygroscopic material such as polyimide. The polyimide as a hygroscopic coating expands due to the absorption of water. Due to the unequal expansion that occurs between the polyimide and polysilicon, a change in the bending structure of the membranes is observed. The change in bending is converted into an electrical signal via a piezoresistor. The hygrometric sensor has some advantages as well as some disadvantages which are listed below:

*Advantages*:
  - Low cost;
  - No power supply is required;
  - It provides a primary rather than a secondary measurement of dew point;
  - High accuracy;
  - High repeatability;
  - Traceable to N.I.S.T. or other national standards labs;
  - Wide dew point range;
  - Wide temperature range;
  - Low hysteresis;
  - No drift.

*Disadvantages*:
  - More complex, may be more expensive;
  - Narrow flow rate range;
  - Periodic cleaning may be required;
  - High uncertainty (±2% to 5%).

### 2.2. Resistive Sensors

**Figure 3.**
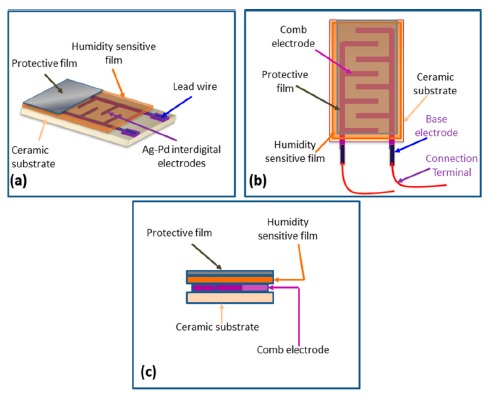
Different views of resistive type humidity sensors: (**a**) isometric view; (**b**) top view; (**c**) cross-sectional view.

The most common resistive sensors are based on electrical resistance. The electrical resistance of a material is the opposition to the passage of an electric current through that conducting material. Since the humidity can change the electrical resistance or impedance of a material noticeably, resistive humidity sensors have been developed using this principle. Generally the all resistive sensors have four layers: substrate, interdigital electrode, humidity sensitive film and protective layer which are shown in Figure 3a–c. In resistive or impedance humidity sensors, air humidity changes with the variation of resistance and follows Equation (2) [11]. Measurement of resistance is very simple and straight-forward in comparison to capacitance measurement. Ceramics, polymers and electrolytes are commonly used material for resistive type sensor:

(2) $$\mathrm{Log}(\frac{R\left(r_{h}\right)}{R_{0}})=\frac{\log a-\text{log}r_{h}^{n}}{1+b/r_{h}^{n}}$$

where *R*(*r_h_*) represents the resistance at relative humidity (RH) at a specific humid condition *r_h_, R*_0_ is the resistance at zero humidity, and *a* and *b* are the constants.

The sensing response (*S_R_*) can be measured using an expression given in Equation (3) [12]:

(3) $$S_{R}=\frac{R_{h}-R_{0}}{R_{0}}\times 100\%$$

where *R*_h_ refers to the resistance at certain humidity and *R*_0_ represents the original resistance of the sensors at air of 3% RH.

A CA-NH_4_BF_4_-PEG_600_ thin film resistive type humidity sensor is based on cellulose acetate (CA), ammonium tetrafluoroborate (NH_4_BF_4_) and polyethylene glycol (PEG_600_) [13]. This sensor shows a very good response towards humidity, good linearity and good stability. The size of the resistive humidity sensor can be reduced by using excellent electrically sensitive multiwall carbon nanotube (MWCNT)-reinforced polyimide NC films [14]. The flexibility of a resistive micro-electrode (1.4 mm × 1.4 mm in size) has been improved by using a polyimide substrate and a commercial alumina-based electrode [15]. The interpenetrating polymer network (IPN) of polyimide enhance the polyelectrolyte and water molecule interactions, which provide high sensitivity, water durability, and stability under high humidity and humidity condition to the micro sensors. Resistive sensors have some advantages as well as disadvantages which are listed below:

*Advantages*:
  - Small and cheap;
  - Mass production possible;
  - Interchangeable/field replaceable.

*Disadvantages*:
  - Limited range (typically 15% to 95% RH);
  - High temperature dependency;
  - Poor stability;
  - Sensitive to contamination, condensation;
  - Reading altered by all substances that affect resistance; salts, hydrogen, oxidizing agents, other chemicals;
  - Resistive type sensors find difficulty in measuring low values (below 5% RH);
  - The change in impedance is too high and hence it is difficult to control the dynamics and temperature effects significantly.

### 2.3. Capacitive Sensors

**Figure 4.**
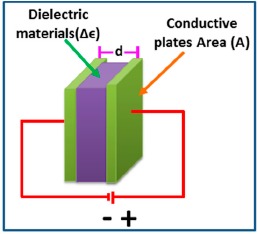
Basic schematic diagram of a parallel plate capacitor.

The capacitance of a sensor indicates the ability of a body to store an electrical charge. It can change significantly with humidity. Using this principle, capacitive type humidity sensors have been developed. Fundamentally, the operation of capacitive type humidity sensors is a function of the dielectric changes of thin films due to the absorption of water vapor. Their characteristics mainly depend on the hygroscopic nature of the material and the geometry of the electrode. In this regards, capacitance (C) of all the parallel plate capacitive sensors (see Figure 4) follows a relation given by Equation (4):

(4) $$\text{C}=\frac{\in A}{d}$$

where ∈ is the dielectric permittivity, A is the overlapping area, and d is the distance between two parallel plates. The sensitivity (*S_c_*) of this capacitive humidity sensor is evaluated using Equation (5) within a certain range of humidity:

(5) $$S_{c}=\frac{\text{maximum capacitance}}{\text{minimum capacitance}}-1$$

A capacitive type humidity sensor based on gold-poly(vinyl alcohol) (PVA) encapsulated gold (Au) nanoparticle nanocomposites (NCs) was analyzed by Yao *et al.* [16]. Here, Au-PVA core-shell NCs were used as dielectric material in between the electrodes. In most capacitive sensors, different types of polymers or elastomer-based materials are used as dielectric materials. The variation of capacitance can be measured as a function of the change in dielectric constant of the polymeric materials, which changes with the absorption of moisture or humidity. On the other hand, silicon (Si) chips and microscopic glass slides are generally used as electrode substrates, while two electrodes are typically coated with conducting metal such as silver (Ag) on the surface of substrate by using advanced nanotechnology. An ultra-thin flexible capacitive humidity sensor was designed by Pantalei *et al.* [17]. This capacitive sensor consists of two parallel metal plates separated by a thin film layer of bis(benzocyclobutene), which behaves as a dielectric material. The two metallic plate electrodes are arranged properly, so that the dielectric material can easily absorb atmospheric moisture or chemical compounds. Wei *et al.* [18] developed a femto-farad capacitive sensor for picoliter liquid monitoring. The sensor consisted of vertical silicon electrodes integrated into a through-wafer channel for the measurement of the liquid level variations inside a channel. Currently, detection of high-resolution capacitance is a big challenge. In this context, Carminati *et al.* [19] have developed a highly accurate and precise calculation of capacitance using a complementary metal oxide semiconductor (CMOS)-based nanosensor. This sensor circuit consists of a CMOS ultra-low-noise and wide-bandwidth current sensing circuit, coupled to a lock-in amplifier for the measurement of capacitance and conductance in a frequency range from DC to 1 MHz. Matko *et al.* [20] highlighted a highly sensitive capacitive humidity sensor for high air humidity measurements. This sensor consists of an analog-to-digital (A/D) to digital-to-analog (D/A) converter and crystal oscillators. Their device showed as a highly sensitivity but the stability of crystal oscillator is still a big challenge. A stability analysis of a crystal oscillator was reviewed by Wall *et al.* [21]. After long time operation of the oscillator, it is normally affected by aging effects [22]. Matko [23] developed a quartz sensor for water absorption measurements in glass-fiber resins (GFRs). The GFRs-based capacitive sensor is not highly precise due to its nonlinear frequency-temperature characteristics, however, this problem was solved by using an AT-cut quartz crystal sensing device [24]. There are several high quality capacitive methods for absorption of water vapour (including without thin films) in humidity measurements which use simple open capacitors (with good electronic circuits and quartz oscillators) in a form of a comb or two plates, and have many advantages and disadvantages such as [18–24]:

*Advantages*:
  - Wide measurement range 0%–100% RH;
  - Wide temperature range (up to 200 °C);
  - Low temperature dependence (temperature compensation);
  - Low hysteresis (below 1%);
  - Low drift;
  - Have no problems with high humidity measurement or saturation and response time;
  - Have no absorbing material;
  - Excellent stability & linearity;
  - Fast response;
  - Full recovery from condensation;
  - Highly resistant to contaminants;
  - Small in size;
  - Low cost;
  - Require very little maintenance.

*Disadvantages*:
  - Can be limited by distance from electronics to sensor;
  - Loss of relative accuracy at low end (<5%);
  - Requires electronics to convert capacitance to relative humidity;
  - Significant drift.

### 2.4. Gravimetric Sensors

**Figure 5.**
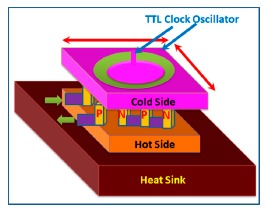
The schematic diagram of a QCM humidity sensor [25].

**Figure 6.**
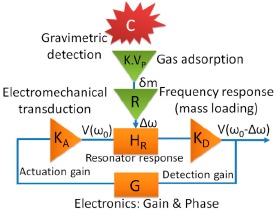
Schematic of a gravimetric sensor based on frequency shift detection [28].

The quartz crystal microbalance (QCM) [25] is the main source of inspiration for gravimetric sensors. This sensor consists of a piezoelectric quartz plate, which is coated with a hygroscopic material having resonance frequency in the MHz range, and it measures humidity due to the change of frequency as shown in Figure 5. The change of frequency (Δ*f*) can be calculated by the Sauerbrey Equation (6) [26]:

(6) $$\Delta f=-2\frac{1}{A}\frac{f_{0}^{2}}{\sqrt{µ\rho }}\Delta m$$

where *A* represents the area of surface, μ is the shear modulus, *f*_0_ is the nominal frequency, ρ is the density, *Δm* is the mass change due to absorption of moisture. Another example of gravimetric humidity sensors is the cantilever type resonator that consists of two electrodes which can be coated with a flexible polymer such as polyvinyldifluorene (PVDF) on both sides [27]. When an electrical signal is applied on both sides of the electrode, the electrode cantilever starts to vibrate due to expansion and compression. Due to the absorption of moisture, a cantilever mass change *Δm* will occur that causes a frequency change in the beam. A general schematic of a gravimetric sensor based on frequency shift detection is illustrated in Figure 6 [28].

In this case, a resonance frequency shift occurs due to the absorption of mass in the chemical layer which is recorded by the resonator. The gravimetric sensor has some advantages as well as some disadvantages which are listed below:

*Advantages*:
  - Relatively low response time;
  - Good accuracy;
  - Low drift.

*Disadvantages*:
  - Nonlinear characteristics;
  - High temperature dependence;
  - Require complicated systems for signal processing of sensor outputs.

### 2.5. Optical Sensors

All the above discussed humidity sensors have their own transduction principles to measure the humidity with some advantages and disadvantages. Normally electronic devices related to the above sensors cannot work under electromagnetic interference or in remote monitoring systems and hazardous or explosive environments. However, optical sensor-based humidity sensors are ideal devices to work under the above severe conditions, thus they sees significant use in many advanced applications where they show enhanced efficiency. The detection limit of this optical humidity sensor is less than 4% RH.

**Figure 7.**
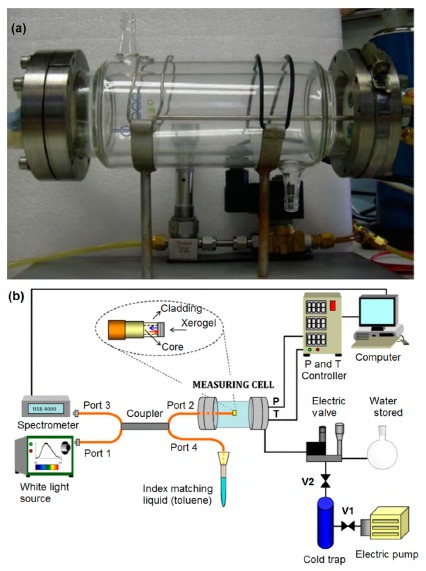
Optical fiber humidity sensor. (**a**) Image of a fiber optic humidity sensor [29]; (**b**) Schematic of an optical humidity sensor [29].

Hence, based on the versatile applications and numerous advantages of optical fiber technology, researchers have been focusing on fiber optic-based techniques for the measurement of humidity for the last few years. A fiber optic humidity sensor proposed by de Vicente *et al.* is depicted in Figure 7a [29]. A schematic of an optical humidity sensor is depicted in Figure 7b. The main sensing principle of an optical humidity sensor is based on the change in reflected optical power due to the water molecules adsorbed on a porous sensing element such as a silica xerogel film, which is embedded on the optical fibers. The relative reflected power at an interface between the two media changes with the refractive index of the media, incidence angle and polarization of the incident wave.

The reflected optical power (*I*) due to the perpendicular light impingement from the interface depends on the refractive indices n_1_ and n_2_ of the two media and follows Equation (7) [29]:

(7) $$I=I_{0}{\left[\frac{n_{2}-n_{1}}{n_{2}+n_{1}}\right]}^{2}$$

where *I*_0_ is the optical power monitored from the reference signal.

A porous sensing film of silica xerogel on the tip of an optical fiber provides an optical cavity where the fiber-xerogel interface gives the first refection and the xerogel-vapour interface gives the second reflection. The reflectance of this sensing element can be expressed by Equation (8) [30]:

(8) $$R=|r{|}^{2}=\frac{r_{FX}^{2}+r_{XV}^{2}+2r_{FX}r_{XV}\cos2\beta }{1+r_{FX}^{2}r_{XV}^{2}+2r_{FX}r_{XV}\cos2\beta }$$

where *r_FX_* is the coefficient of reflectivity at the fiber-xerogel interface, which depends on the effective refractive indices of the fundamental mode of optical fiber (*n_eff_*) and the film (*n_f_*) (see Equation (9)); *r_XV_* the coefficient of reflectivity at the xerogel-vapour interface depends on the *n_f_* and the refractive index of the external medium (*n_em_*) (see Equation (10)) and β is directly proportional to *n_f_* and the film thickness (d) and inversely proportional to the optical wavelength (λ) (see Equation (11)).

(9) $$r_{FX}=\frac{n_{\text{eff}}-n_{f}}{n_{\text{eff}}+n_{f}}$$

(10) $$r_{XV}=\frac{n_{f}-n_{em}}{n_{f}+n_{em}}$$

(11) $$\beta =\frac{4\pi n_{f}}{\lambda }d$$

According to Equations (7) and (8), it is obvious that the variation in refractive index of the sensing film (xerogel) and the external medium leads to change in the reflectance at the fiber-film interface and, therefore, in the sensor output signal. The changes in the reflectance at the fiber-film and film-external medium interfaces also vary with film thickness. Optical fiber humidity sensors have many advantages compared with conventional measuring methods, but also some disadvantages, which are given below:

*Advantages*:
  - Low cost;
  - High sensitivity;
  - Small size;
  - Robustness;
  - Flexibility;
  - Ability for remote monitoring as well as multiplexing;
  - Used even in the presence of unfavorable environmental conditions such as noise, strong electromagnetic fields, high voltages, nuclear radiation, in explosive or chemically corrosive media, at high temperatures;
  - Good reproducibility;
  - Low hysteresis.

*Disadvantages*:
  - Inherent losses;
  - Dispersion;
  - Nonlinearity;
  - Birefringence.

There are various types of fiber optic humidity sensors to fulfill the desired advanced needs, such as fiber grating sensors, evanescent wave sensors, interferometric sensors, hybrid sensors (*i.e.*, grating integrated with interferometric), absorbance sensors, direct spectroscopic-based sensors and fluorescence-based sensors *etc.* and listed in Table 1, which covers all types of optical humidity sensors together to highlight the potential results of some reported works chronologically.

### 2.6. Mechanical-Optoelectronic Humidity Sensors

**Figure 8.**
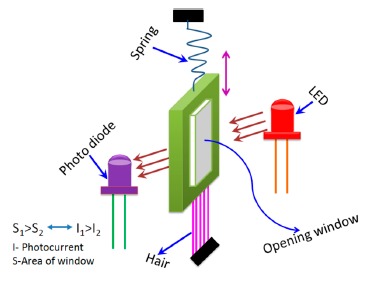
Schematic representation of a humidity sensor that operates by the mechanical-optoelectronic principle.

In addition to the abovementioned five main types of humidity sensors, they can also be categorized by principle, which is the mechanical-optoelectronic principle, as shown in Figure 8 [72]. This device consists of a light emitting diode (LED), a very sensitive photodiode, and a mechanical system. The sensor has a bunch of human hair at one end and other end has a thin metal sheet with a fittable window with respect to the LED and photodiode. Human hair acts as a capacitor in this humidity sensor. A spiral spring is connected with the metal sheet. When the humidity concentration changes the contraction and expansion occurs in the hair, this hair pulls the metal sheet up or down. Thus, the window opening area changes and this varies the light intensity reaching the photo detector from the LED, resulting in a photocurrent change with respect to humidity on the output side. This sensor has a very good linearity, long life time, small hysteresis, stable operation, and less temperature dependency.

## 3. Different Key Sensing Materials and Their Characteristics in Humidity Sensors

During the last five decades, plenty of sensing materials have been developed for different types of humidity sensors in a wide range of applications. The most common commercial sensors are mostly based on metal oxides, porous silicon and polymers [12]. In a humidity sensor, fundamentally, the humidity signals obtained via two-electrode techniques are affected by polarization effects. This happens due to the migration of electrons from the metal probe into the conductive specimen. Polarization effects may be caused by the migration of H^+^ ions into the metallic probe as H^+^ ions are found in the physisorbed absorbed water [12,73]. Thus, positive charges in the specimen migrate towards the metallic probe and create a cation layer between the metallic probe and the specimen. In this context, plenty of electrode structures have been proposed for various sensing systems. Hence, this review article is mainly focused on the key sensing materials and their typical characteristic properties for the application in humidity sensors.

Addition of different coatings or dopants to a base material changes its physical, chemical, electrical as well as structural characteristics and these characteristics are also changed with different base materials. In this section, different electrical characteristics (*i.e.*, impedance or resistance, capacitance, response and recovery time, hysteresis, and stability with the variation of relative humidity, frequency, and temperature) of various sensor materials based on carbon, vanadium, iron, silicon, polymer, tin, titanium, zinc, zirconia and sodium are compared. Both similarities and dissimilarities in the electrical characteristics for different sensor materials are observed, which vary strongly with the doping concentration of different materials, film thickness of the substrates and the morphological changes.

Although humidity sensors based on different materials have been used for more than six decades, the key factors to improve sensing characteristics in different applications are still not well defined. This is extremely important in the miniaturization of sensors in nanotechnology for many advanced applications. Therefore, in ordered to determine the best humidity sensor based on different materials, the plots of the important electrical characteristics of various morphological structures, coatings or doping agents are recognized and typically compared.

### 3.1. Carbon

**Figure 9.**
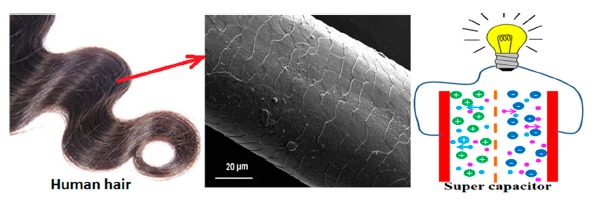
Schematic representation of human hair-derived carbon used as a supercapacitor.

Carbon is highest abundance material present on Earth. Different allotropic forms of carbon have been widely used in broad application fields. Carbon materials are available in different forms like carbonized porous form [74], graphite [75], diamond or diamond like carbon [76], fullerenes [77,78], graphene [79], amorphous carbon [79–81], carbon nanotubes (CNTs) [82], carbon nanofibers (CNFs) [83] and nanostructured carbon films (carbon nanosheets and nanohoneycombs) [12]. A small amount of carbon present as an inclusion or dopant may also be able to change the properties of materials exponentially. Interestingly, human hair-derived carbon flakes have recently shown excellent capacitance behaviour and thus have been used as a supercapacitor (Figure 9) [84].

Carbon nanostructured materials have shown the most attractive sensing properties in various applications. A large sensing area, high chemical inertness, and large porosity nature of carbon make it popular in the humidity sensor field. Different techniques such as physical vapor deposition, thermal carbonization, magnetron sputtering, thin film deposition and so on have been used to fabricate carbon-based nanostructured humidity sensors. The doping of different materials in carbon changes its physical, electrical and humidity sensing characteristics as explained below. The electrical characteristics (resistance, capacitance, sensitivity, response time, hysteresis, stability) changes with the variation of frequency, temperature and relative humidity of carbon-based sensor materials are explained in the following subsections.

#### 3.1.1. Effect of Temperature and Frequency on Resistance-RH Characteristics

**Figure 10.**
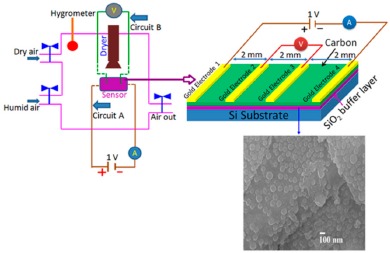
Nanostructured carbon film (carbon nanosheets and nanohoneycombs) coated on Si(100) substrate in a humidity sensor device (adopted from [12]).

For carbon-based resistance humidity sensors, in most cases it has been found that the resistance increases as the RH value increases. The change in resistance is greater in the high humidity region. Nanostructured carbon films (*i.e.*, carbon nanosheets and nanohoneycombs) coated on Si(100) substrate have been used in a humidity sensor device, which is depicted in Figure 10 [12].

**Figure 11.**
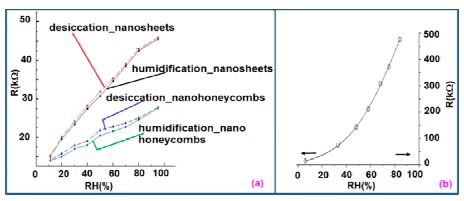
(**a**) Linear variations of resistance with RH for carbon nanosheet- and nanohoneycomb-based sensors due to both desiccation and humidification [12]; (**b**) Nonlinear behaviour of resistance with RH for a (TC-PS)-based humidity sensor [74].

It shows a linear variation of resistance with relative humidity in the range of 11%–95% as illustrated in Figure 11a [12]. The resistive sensing response of the carbon nanosheet- and nanohoneycomb-based sensors increases up to 225% and 110%, respectively, at 95% relative humidity (RH). It has also been found that nanocarbon films, including carbon nanosheet- and carbon nanohoneycomb-based sensors give excellent linearity (see Figure 11a) under any humidity condition between 11% and 95%. Unlike nanocarbon films, thermally carbonized porous silicon (TC-PS)-based humidity sensors show nonlinear behavior in their resistance *vs.* RH characteristics with a significantly higher resistance value (see Figure 11b). The non-linearity of the TC-PS based humidity sensor is related to the dielectric changes of a porous layer upon water vapor uptake while the condensed vapor replaces the air in the pores as the relative humidity increases [74]. It is to be noted that linearity in this characteristic is more desirable to improve the efficiency of sensors. The resistance of the carbon-based sensors also strongly depends on the temperature as well as structural morphology. The resistance value decreases with increasing temperature owing to the increased amount of free electrons. Chu *et al.* showed that the resistance of nanohoneycomb carbon is significantly lower than that of nanosheet carbon when all other conditions are the same [12]. This may be due to more transportation freedom for the migrating ions or free charges available in the nanohoneycomb structure compared to nanosheets. In this context, a quantitative analysis showed that a charge transfer of 0.03 e^−^ from a single water molecule to a carbon nanofilm can occur [12,85]. It has also been reported that the variation of frequency may affect the resistance value. In a multiwall carbon nanotubes (MWCNTs)-based humidity sensor, the resistance decreases with increasing frequency [86].

#### 3.1.2. Effect of Frequency on Capacitance-RH Characteristics

**Figure 12.**
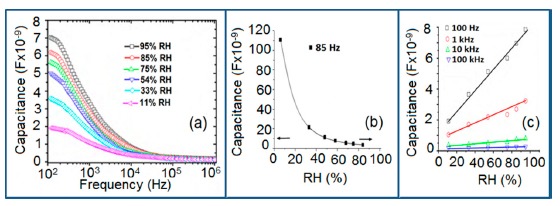
(**a**) Nonlinear behavior of capacitance with frequency of a-C/n-Si junction as a function of RH at 6 Pa and room temperature [87]; (**b**) Variation of capacitance with RH range (11%–95%) for a TC-PS based humidity sensor at a constant frequency [74]; (**c**) Linear behavior of capacitance with RH range (11%–95%) for a-C/n-Si-based humidity sensor at different constant frequencies [87].

The capacitance value of a humidity sensor generally increases nonlinearly with increasing relative humidity. This has been clearly observed over a wide range of RH values from 11% to 95% for a capacitive humidity sensor in which amorphous carbon (a-C) films are deposited on nanosilicon (n-Si) [87]. Here, the a-C and Si form a heterojunction layer. The capacitance of the heterojunctions increases with decreasing frequency at high RH values and this is depicted in Figure 12a.

a-C/n-Si sensors have shown excellent linearity in comparison to TC-PS-based humidity sensors. The range of capacitance values in a-C/n-Si based sensor is several times higher than in TC-PS-based humidity sensors, however, it has also been found that the capacitance decreases with increasing RH for TC-PS material [74]. This is related to the dielectric changes of a porous layer upon water vapor uptake when the air in the voids is replaced by condensed vapor as the relative humidity increases. The capacitance variations with different RH values for both the sensors are shown in Figure 12b,c. For practical applications, a-C/n-Si based sensors give optimized results at 1 kHz frequency since there they show the best linearity as compare to other frequencies [87].

#### 3.1.3. Response and Recovery Time

For the estimation of the performance of humidity sensors, response and recovery behavior are some of the most significant features. Response time is defined as the time taken by the sensor to achieve 90% of the total resistance or capacitance change in case of adsorption and the recovery time in case of desorption of the water vapors. The response and recovery time are very structure sensitive. The values are different for different carbon material morphological structures in humidity sensors. The response time of a TC-PS-based capacitive humidity sensor was 90 s in a low humidity range (<70% RH), but at higher RH (>90%) the response time was 120 s [74], whereas, the response time of a MWCNTs-based humidity sensor was 16 s and the recovery time was 8 s [86]. The response and recovery time of a-C/n-Si based sensor were 3 min and 4 min, respectively [87]. The response and recovery times of a carbon nanosheet-based sensor were 30 s and 90 s, respectively, at 40% RH, which are even better than that obtained with a carbon nanohoneycomb-based sensor [12]. This has been observed because the nanoparticles on the surface of the carbon nanosheets make water molecules adsorb more easily compared to carbon nanohoneycombs. The hydroxyl groups in the water molecules act as adsorption sites for water molecules to connect between the nanoparticles on surface of the carbon nanostructured materials. For a practical application, the response and recovery time of a sensor must be as short as possible. For comparison, it is concluded that the MWCNTs-based humidity sensor has the smallest response and recovery time and the a-C/n-Si based sensor has the highest response and recovery time. The a-C/n-Si based humidity sensor may give excellent linearity but it has very slow response for humidity sensing, therefore, a MWCNTs-based humidity sensor would be a first choice as best humidity sensor.

#### 3.1.4. Sensitivity Response

The humidity sensitivity is determined from the slope of a capacitance *vs.* RH curve. The sensitivity of capacitive and resistive type humidity sensors can be calculated using Equations (3) and (5), respectively. The sensitivity of a humidity sensor can also be evaluated at different frequencies. It has been reported that the sensitivity of a TC-PS sensor at 55 Hz, 120 Hz and at 1 kHz is 900%, 800% and 450%, respectively [74]. The sensitivities of a-C/n-Si sensor at 100 Hz, 1 kHz, 10 kHz, and 100 kHz are reportedly 0.9887, 0.9470, 0.8484, and 0.7881, respectively [87]. It has been noticed that the sensitivity decreases with increasing frequency and the sensors give maximum sensitivity at the low frequency range. Like the capacitance sensitivities, conductance sensitivity can also be calculated to evaluate a sensor using Equation (12) at minimum and maximum RH vales [86]:

(12) $$\text{Conductance sensitivity}=\text{G}{\left(\omega \right)}_{97\%}-\text{G}{\left(\omega \right)}_{11\%}$$

where the G(ω) terms represent the frequency dependent conductance at the maximum (e.g., 97%) and minimum RH values (e.g., 11%).

It has been reported that the conductance sensitivity of a MWCNTs-based sensor is −0.02 ms at 50 Hz and at 1 MHz the sensitivity (S) reaches its maximum value (1.906 ms) [86]. This indicates that the conductance sensitivity increases as the frequency increases but the capacitance sensitivity decreases with increasing sensitivity.

#### 3.1.5. Hysteresis Characteristics

Hysteresis is defined as the non-coincidence between the loading and unloading behavior of a particular plot. The loading and unloading curves of a perfect sensor normally follow the same path, which is different for different morphologies. For example, the maximum humidity hysteresis for nanostructured carbon sensors such as carbon nanosheet- and nanohoneycomb-based sensors is 3.57% and 6.83%, respectively, at 50% RH [12]. Although nanohoneycomb morphology carbon films show better properties in resistive and capacitive humidity sensors, from a hysteresis point of view carbon nanosheet humidity sensors are more suitable for humidity sensing applications than carbon nanohoneycomb-based sensors since a lower hysteresis value is more desirable for stabilization of an electronic instrument. On the other hand, TC-PS-based humidity sensors show poor hysteresis, even at different frequencies [74], although increasing the porosity may improve the hysteresis by widening the porosity in the material structure. However, while this technique may help to reduce the hysteresis the sensitivity and response time of the TC-PS sensor may suffer significantly.

#### 3.1.6. Stability and Repeatability Analysis

**Figure 13.**
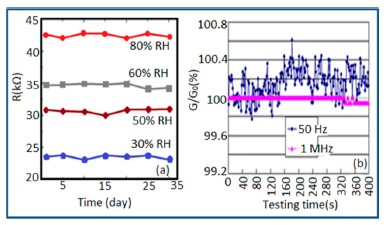
(**a**) Stability analysis for a carbon nanosheet-based humidity sensor at different % RH values; (**b**) stability analysis for a MWCNTs-based humidity sensor at different frequencies [86].

Carbon nanofilm-based sensors showed good stability and durability in a repeatability study when tested for a month at 5-day intervals, as shown in Figure 13a [12]. It has also been noticed that at high frequency range, nearly 1 MHz, MWCNTs-based humidity sensors show greater stability compared to a lower frequency such as 50 Hz, as depicted in Figure 13b [86].

**Figure 14.**
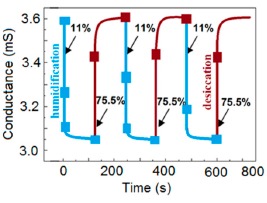
Repeatability analysis for MWCNTs-based humidity sensor materials.

Furthermore, TC-PS-based sensors have shown less stability compared to carbon nanosheet- and MWCNTs-based sensors. On the other hand, for MWCNTs sensors, an excellent reversibility or repeatability in conductance with RH through adsorption-desorption dynamic cycles at 1 MHz has been repeatedly shown between 11% and 75.5% RH, as illustrated in Figure 14 [86]. Similar comparisons in repeatability of capacitance-time plots between two different materials such as a-C/n-Si (at 1 kHz, between 33% and 95% RH) [87] and TC-PS (at 120 Hz, between 6% and 58% RH) [74] have also been reported. This comparison indicates that the TC-PS based sensor shows repeatability at low humidity range at low frequency, which is also lower than that of MWCNTs-based humidity sensors (which showed repeatability at 1 MHz).

### 3.2. Vanadium-Based Materials for Humidity Sensors

The abrupt change in electric properties and a sharp change in optical transmittance or reflectance make vanadium (V) a “smart material” [88,89]. Vanadium has a tremendous application in the fields of thermochromic coatings, temperature sensing devices, optical switching devices and Mott field-effect transistors [90,91]. Ceramic materials have potential applications in the field of humidity sensors. Recently rutile phase vanadium dioxide (VO_2_) (R/M_1_) has also been used in humidity sensors [91] since it undergoes a first-order reversible metal-insulator transition (MIT) near room temperature (68 °C) [92] among over ten crystalline phases of VO_2_ [93]. Nanostructured VO_2_ has received great attention owing to its physical and chemical properties that differ greatly from those of the bulk counterpart [91]. Vanadium-based humidity sensor materials such as nanostructured VO_2_(B) or VO_2_(M_1_) can be synthesized by several methods such as thermolysis [94], solution-based methods [95], hydrothermal methods [91], and hydrothermal methods followed by heat-transformation [91], sol-gel [96], chemical vapor deposition [97], pulsed laser ablation [98], magnetron sputtering [99], and so on. The morphology of VO_2_ can be changed by using different synthesis techniques. It has been found that different concentrations of reductant also can change the morphology of VO_2_. Recently, Yin *et al.* [91], have shown that with variation of a vanadium salt concentration (*i.e.*, NH_4_VO_3_ as a source) in a reductant (*i.e.*, oxalic acid) the morphology of nanostructured VO_2_(M_1_) could be changed to nanospheres integrated with nanowires (synthesized with 0.125 mol/L oxalic acid at 180 °C for 24 h), nanoflowers (synthesized with 0.1 mol/L oxalic acid at 180 °C for 24 h) and nanorods with some nanoflakes (synthesized with 0.065 mol/L oxalic acid at 180 °C for 24 h). The all the basic properties, including field emission (FE) current density (J, mA/cm^−2^) and sensitivity of the nanostructured VO_2_(M_1_) based humidity sensors changed significantly with the different structures. The variations of the electrical characteristics (resistance, capacitance, sensitivity, response time, hysteresis, stability) of vanadium-based sensor materials with the variation of frequency, temperature and relative humidity are explained in the following subsections.

#### 3.2.1. Field Emission Characteristics

**Figure 15.**
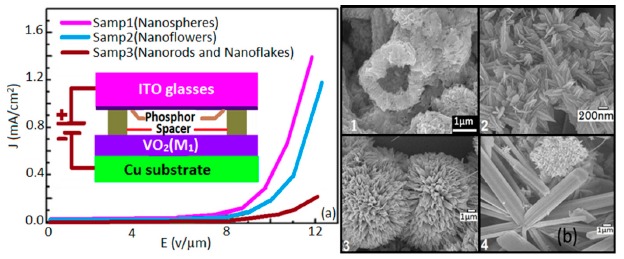
Structural morphology and electric field response of vanadium-based materials. (**a**) The field emission current density (J) dependence on applied electric field for nanospheres (Sample 1), nanoflowers (Sample 2), and nanorods and nanoflakes (Sample 3); (**b**) Scanning electron micrographs of the nanospheres (1), nanoflakes (2), nanoflowers (3), and nanorods and nanoflakes (4) [91].

The field emission current density J increases exponentially with the applied field (E, V/μm) and the nanosphere structure. As depicted in Figure 15a, a Sample1-based sensor showed the highest FE current with the best turn-on field (around 4 V/μm) when the threshold field is the lowest (around 11 V/μm). The turn-on (around 5.3 V/μm) and threshold (may be more than 13 V/μm) fields are the poorest for the nanorods with some nanoflakes-based sensors (Sample 3).

Emitter geometry (such as aspect ratios), crystal structure, and the spatial distribution of emitting centers are the main cause for this field emission effect. Each nanosphere Sample1 sensor has many VO_2_(M_1_) nanowires or belts (nearly 100 nm wide) with a large aspect (*i.e.*, length-to-diameter) ratio which easily enhances the electron emission from these nanowires (see Figure 15b). On the other hand, Sample 3 has large diameter nanoroads without having any sharp tip, which is the main reason of the electron emission. The blunt edged larger diameter structures in Sample 3 causes its less electron emission. Furthermore, VO_2_(M_1_) nanoflowers and hollow nanospheres both are more sensitive to humidity since both have high surface to volume ratio.

#### 3.2.2. Effect on Resistance of the Variation of RH

**Figure 16.**
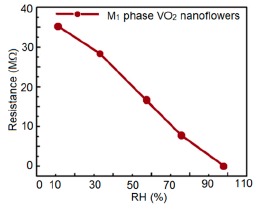
Variation of resistance with relative humidity for VO_2_(M_1_) nanoflowers.

In general vanadium-based humidity sensor materials show linear resistance characteristics. The resistance decreases with increasing RH, as shown in Figure 16. In contrast, water coverage on the surface of VO_2_(M_1_) nanoflower structured sensors is not continuous under low humidity conditions, which causes a very weak electrolytic conduction. Thus, the resistance value becomes high since the sensor surface absorbs very few water particles discontinuously. On the other hand, under high humidity conditions, a number of continuous water layers are formed on the VO_2_(M_1_) porous structure, which causes a very good electrolytic conduction by accelerating H^+^ ions and thus, the value of resistance decreases.

#### 3.2.3. Sensitivity Analysis

Sensitivity is measured from the slope of the linear fit resistance *vs.* RH curve. The sensitivity factor (S_f_) is the ratio of minimum RH to maximum RH. The VO_2_(M_1_) nanoflower-based humidity sensors show extremely high sensitivity, *i.e.*, 118.

#### 3.2.4. Response and Recovery Characteristics

Response and recovery times are the most important dynamic characteristics of all sensors. VO_2_(M_1_) nanoflower-based humidity sensors show a response time of 13 s at 1 V AC signal and frequency of 100 Hz and the recovery time is 5 s. The VO_2_(M_1_) nanoflower-based humidity sensors have better response compared to the ZnO- [100], SnO_2_- [101], and TiO_2_-based [102] resistive sensors.

#### 3.2.5. Stability and Reproducibility Analysis

**Figure 17.**
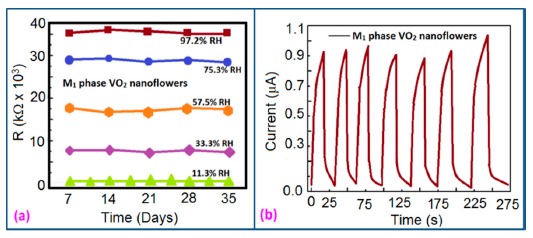
(**a**) Stability analysis and (**b**) reproducibility analysis curves of vanadium oxide-based materials.

Stability and reproducibility are also very important dynamic characteristics of a sensor. Yin *et al.* [91] have done a stability analysis of VO_2_(M_1_) nanoflower-based humidity sensors by repeatedly measuring the resistance once a week for five weeks by exposing the sensor to air. The resistance fluctuations showed by this sensor was less than ±10% over the five weeks, which shows excellent stability (Figure 17a) and good reproducibility (see Figure 17b) with seven consecutive cycles with narrow fluctuation. This narrow fluctuation occurred due to the fluctuation of RH in the laboratory atmosphere and the moist air which was formed in an aqueous solution of K_2_SO_4_.

### 3.3. Iron-Based Materials for Humidity Sensors

Ceramic materials have tremendous applications in the field of humidity sensors. The most commonly used ceramic materials for humidity sensors are TiO_2_, ZnO, ferrite, silica, perovskite oxides BaTiO_3_, and (Ba,Sr)TiO_3_. Copper-zinc spinel ferrite has a very high electrical resistance, due to which it can't be used as a good sensitive material for humidity sensors. To bring this ferrite material into a measurable zone and to increase its conductivity, tungsten and iron have been added to this composite [103] to develop a copper-zinc-tungsten Cu_0.5_Zn_0.5_W_0.3_Fe_1.7_O_4_ spinel ferrite by the sol-gel technique. On the other hand, maghemite γ-Fe_2_O_3_ is an n-type semiconductor and many researchers have used it as a humidity sensor material. γ-Fe_2_O_3_ based sensor has significantly high sensitivity, simple design, and low cost, but this sensor material lacks selectivity. This problem can be overcome by different doping techniques using different ions such as lithium (Li^+^) [104], tin (Sn^4+^) [105], molybdenum (Mo^6+^) [105] in iron based humidity sensors. In contrast, lithium has been doped in γ-Fe_2_O_3_ based humidity sensors by a chemical synthesis technique [104] and Sn^4+^ and Mo^6+^ have been doped by substituting Mg and/or Fe in MgFe_2_O_4_ to produce a potential humidity sensor composite material (*i.e.*, Mg_1−_*_x_*Sn*_x_*Fe_2−_*_y_*Mo*_y_*O_4_) using the sol-gel technique [105]. Many other humidity sensor materials have also been developed using different synthesis techniques. Li-doped iron oxide thin film-based humidity sensors have been developed using a liquid phase decomposition technique [106]. A potassium (K^+^)-doped LaCo_0.3_Fe_0.7_O_3_ thick film humidity sensor was developed by using a screen printing technique [107]. It has been found that K-substitution at La-sites in the La_1−_*_x_*K*_x_*Co_0.3_Fe_0.7_O_3−δ_ perovskite structure has a significant effect on humidity detection [108]. The perovskite structured composite developed by the sol-gel technique has shown better homogeneity results and more significant properties compared that prepared by a simple mechanical mixing method. Furthermore, a mesoporous LaFeO_3_ humidity sensor has been developed through a nanocasting method by using mesoporous SBA-15 as a hard template [109]. The high surface area, high pore volume and crystalline wall are the major characteristics of an iron-based humidity sensor. The electrical characteristics (resistance, capacitance, sensitivity, response time, hysteresis, stability) variation with the variation of frequency, temperature and relative humidity of iron-based sensor materials are explained in the following subsections.

#### 3.3.1. Resistance or Impedance Variation with Relative Humidity (RH)

**Figure 18.**
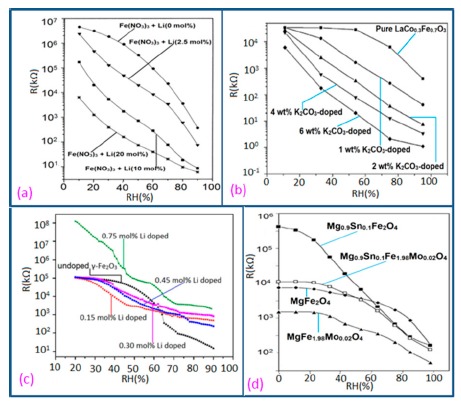
Variation of the resistance with the RH of different iron-based doped materials: (**a**) Li-doped iron oxide thin films [106]; (**b**) K^+^-doped nanocrystalline LaCo_0.3_Fe_0.7_O_3_ [107]; (**c**) Li doped and undoped γ-Fe_2_O_3_ samples [104]; (**d**) Mg^2+^ and Fe^3+^ of MgFe_2_O_4_ with Sn^4+^ and Mo^6+^ [105].

It has been found that a small size and highly local charge content Li-ions significantly enhance the water absorbing property in a sensor, which leads to more chemisorbed water molecules on the material surface and lesser resistance in the sensor. Thus, an undoped sensor (FF80) shows extremely high resistance values in comparison to a Li-doped iron oxide sensor. With increasing Li-content in the iron oxide-based humidity sensor the resistance values decrease significantly and at higher RH (>60% RH), the rate of resistance fall is sharp in comparison to low RH range (see Figure 18a) [106]. Like Li^+^ ion, in K^+^-doped LaCo_0.3_Fe_0.7_O_3_ thick film sensors, the resistivity decreases with increasing humidity over the whole range of RH values, as shown in Figure 18b [107]. It is to be noticed that in undoped conditions, in a γ-Fe_2_O_3_-based humidity sensor, the resistance decreases immediately with increasing RH value, but a LaCo_0.3_Fe_0.7_O_3_ thick film sensor is only sensitive to the moisture above 54% RH. Only 2 wt % of K^+^ addition to a LaCo_0.3_Fe_0.7_O_3_ thick film sensor can give excellent resistance linearity with increase of RH. On the other hand, a Li-doped iron oxide sensor does not show any linearity at all. Due to the addition of Li-ions to iron oxide no microstructural changes occur but due to the addition of K^+^ ion in LaCo_0.3_Fe_0.7_O_3_ its pore diameter changes, which in turn changes its resistance characteristics. In both cases, the resistance measured at RH 10% is much lower (up to four orders of magnitude) than that of the undoped films. It has also been reported that the resistance value may increase above a critical amount of doping agent in iron oxide-based sensors.

In contrast, Kadam *et al.*, had shown that 0.45 mol % of Li-doping in a γ-Fe_2_O_3_-based humidity senor is critical, since it showed maximum linearity [104]. Below that level (e.g., 0.15 and 0.30 mol %) this critical doping value showed nonlinear resistance-RH characteristics and above it (*i.e.*, 0.75 mol %), it showed increased resistance values, which were also higher than that of an undoped γ-Fe_2_O_3_-based humidity sensor (see Figure 18c) [104].

Therefore, excess doping, above an optimal or critical value, is not at all suitable for humidity sensor design. Another noteworthy point is that the resistance of Li-doped γ-Fe_2_O_3_ sensors does not decrease continuously over the whole range of RH values like other sensors. It has been reported that tin (Sn)-substituted MgFe_2_O_4_ based ferrite (*i.e.*, Mg_0.9_Sn_0.01_Fe_2_O_4_ sample) showed a higher value of resistivity (10^9^ Ω·cm at 11% to 85% RH) compared to Mo-substituted ferrite (*i.e.*, a MgFe_2_Mo_0.02_O_4_ sample) that exhibits the smallest resistivity (10^6^ Ωcm at 11% to 85% RH (see Figure 18d) [105]. Material composition, crystallite size, surface area and porosity are responsible for humidity sensitivity enhancement in this sensor material. High surface area and large porosity materials absorb more water vapors, which cause a decrease of the resistance value. Sn-substituted ferrite material is the most sensitive material to humidity changes and its resistance deceases with increased humidity, because of its very large surface area (23.8 m^2^/g).

#### 3.3.2. Effect of Frequency on Resistance-RH Characteristics

**Figure 19.**
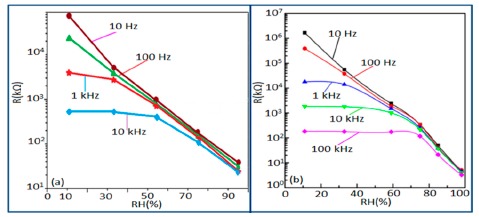
RH dependence of resistance at various frequencies for different iron-based materials. (**a**) La_0.93_K_0.07_Co_0.3_Fe_0.7_O_3−δ_-based humidity sensor; (**b**) mesoporous LaFeO_3_ [109].

Normally in the low frequency region (10 and 100 Hz), iron-based sensors show high sensitivity and good linearity of their resistance-RH or impedance-RH characteristics but at higher frequency, the sensor shows less sensitivity at a low RH range. This effect is observed because at high frequency the absorbed water cannot be polarized, and the dielectric phenomena disappear. Therefore, to get high sensitivity and good linearity a low working frequency is always applicable. For example, with 2 wt % of K^+^ addition in a LaCo_0.3_Fe_0.7_O_3_ thick film sensor, the resistance decreases effectively as the frequency increases at a low RH range and the difference in resistance value between two working frequencies becomes smaller at a high RH range [107]. Similarly, Wang *et al.* [108] observed almost same effect for the impedance with variation of frequency over the whole humidity range by substituting K-ions at La-sites of La_1−_*_x_*K*_x_*Co_0.3_Fe_0.7_O_3−δ_ perovskite for humidity detection as shown in Figure 19a as in another study performed in 2007 [107]. The value of impedance decreases with increasing frequency the low humidity region and in the high relative humidity region the impedance becomes independent of frequency. Good linearity and high sensitivity are observed in the 10–100 Hz frequency range. It has also been noticed that in the low relative humidity region, the frequency has a strong effect on impedance. For example, in the 11% to 98% RH range and at 10 Hz frequency, the impedance of a mesoporous LaFeO_3_ sensor decreases greatly, by more than five orders in magnitude (1.7 × 10^6^ to 4.5 kΩ) with good linearity (see Figure 19b) [109]. The highest humidity response and the best linearity are observed at 10 Hz frequency in the entire humidity range but in the previous two sensors 100 Hz is the best optimized frequency for practical applications. It has also been observed that the impedance of the bulk LaFeO_3_ sensor changes by up to three orders in magnitude at 10 Hz frequency in comparison to mesoporous materials [109]. The mesoporous LaFeO_3_ sensor has much higher response than that of the bulk LaFeO_3_ sensor, probably due to the increased specific pore surface area provided by the mesoporous structure.

#### 3.3.3. Effect of Frequency on Capacitance-RH Characteristics

**Figure 20.**
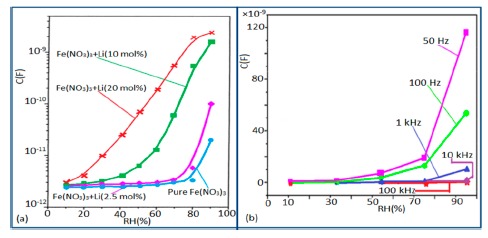
Capacitance variation with relative humidity at different frequencies and with different doping agents in iron-based humidity sensors: (**a**) with different concentrations of Li^+^; (**b**) K^+^-doped iron-based humidity sensor at different frequencies.

In a wide range of RH values, the capacitance values of undoped iron oxide sensors remains constant (*i.e.*, in the range of 10^−11^–10^−12^ F). The capacitance value increases with increased Li doping concentration in iron oxide film over the full RH range at 1 kHz frequency and at high RH (90%), the capacitance value increased by about one order in magnitude (see Figure 20a) [106]. On the other hand, due to the addition of K^+^-ion, the capacitance value of a La_0.93_K_0.07_Co_0.3_Fe_0.7_O_3−δ_-based humidity sensor increases with increasing RH (especially at RH > 54%) in the low frequency range (*i.e.*, ≤100 Hz) and in the high frequency region (*i.e.*, ≥10 kHz), the capacitance becomes almost independent of RH (see Figure 20b) [107].

Due to the addition of K^+^-ion, in the low frequency range (50–100 Hz), the value of capacitance increases with the increasing value of RH (especially at RH > 54%) and in the high frequency region (10–100 kHz), the capacitance becomes almost independent of RH, is illustrated in Figure 20b. In the low frequency region, absorbed water molecules are polarized rapidly and more water particles are absorbed on the surface of the sensor. However, on the other hand, in the high frequency range, the electric field changes its direction rapidly and thus it can't synchronize with the water molecule polarization. As a result, the capacitance value becomes small and independent of RH.

#### 3.3.4. Response and Recovery Time Analysis

**Figure 21.**
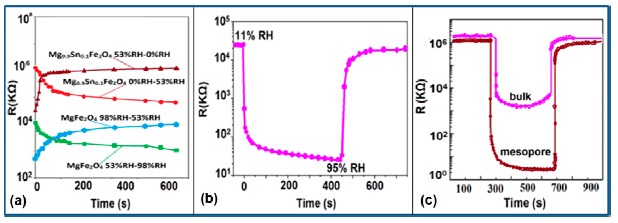
Response and recovery characteristics of different iron-based humidity sensor materials: (**a**) MgFe_2_O_4_ and Mg_0.9_Sn_0.1_Fe_2_O_4_ samples; (**b**) La_0.93_K_0.07_Co_0.3_Fe_0.7_O_3−δ_-based humidity sensor; (**c**) mesoporous and bulk LaFeO_3_ sensor.

Response and recovery characteristics are some of the most significant parameters for estimating humidity sensor performance. It has been found that the addition of Sn^+^ ion may improve the response time of an iron-based humidity sensor. The response times of MgFe_2_O_4_ (in the 53%–98% RH range) and Mg_0.9_Sn_0.1_Fe_2_O_4_ (in the 0%–53% RH range) sensors, which are 5 min and 3 min, respectively, is shown in Figure 21a [105]. For K^+^ doping in an iron-based humidity sensor, the response and recovery times in a humidity range of 33%–95% RH are 100 s and 120 s, respectively [107]. Recently, the response and recovery time resulting from the substitution of K^+^-ion at La-sites of a La_1−_*_x_*K*_x_*Co_0.3_Fe_0.7_O_3−δ_ perovskite humidity sensor have been observed to be 32 s and 50 s, respectively, in the 11%–95% RH humidity range (see Figure 21b) [108].

This comparison result clearly indicates that the K^+^-ion doped perovskite structure [109] of an iron-based humidity sensor has a better response and recovery time compared to a K^+^-ion doped perovskite nanocrystal structure [107] and a Sn-doped [105] sensor. Recently, a response and recovery time comparison for the mesoporous and bulk LaFeO_3_ humidity sensors has been reported by Zhao *et al.*, in which the response times of both sensors was the same, *i.e.*, 1 s, but the recovery time of the mesoporous sensor (*i.e.*, 148 s) is higher than that of the bulk sensor (*i.e.*, 36 s) (see Figure 21c) [109]. In another Li^+^-ion doped γ-Fe_2_O_3_ humidity sensor, the response and recovery time of the sensor were 150 s and 180 s, respectively, in the 20%–90% RH humidity range [104].

#### 3.3.5. Hysteresis Response Characteristics

**Figure 22.**
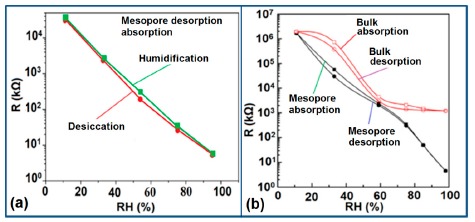
Hysteresis characteristics of different iron-based materials: (**a**) 2 wt % K_2_CO_3_-doped sample with LaCo_0.3_Fe_0.7_O_3_; (**b**) mesoporous and bulk LaFeO_3_ sensor [109].

K^+^-ion-doped iron oxide perovskite type nanocrystals show narrow hysteresis loops during cyclic humidity operation. The hysteresis of a 2 wt % K_2_CO_3_-doped sample between humidification and desiccation processes over the whole humidity range with 1 V, 100 Hz and at room temperature is shown in Figure 22a [107]. A maximum difference in impedance between the adsorption and desorption curves of the La_1−_*_x_*K*_x_*Co_0.3_Fe_0.7_O_3−δ_ perovskite humidity sensor is observed [108]. This sensor exhibits a narrow hysteresis loop with the maximum hysteresis around 4% RH during cyclic humidity operation. For LaFeO_3_ sensors, the largest humidity hysteresis for the sensors using the mesoporous and bulk LaFeO_3_ were observed as 4% and 16%, respectively (see Figure 22b) [109].

Interestingly, in a 0.45 mol % Li doped humidity sensor [104], there is an absence of hysteresis loop during cyclic operation from 20% to 60% RH, but a very narrow hysteresis loop is observed in the humidity range from 65% to 90% RH. After analyzing the above comparison it is confirmed that the bulk LaFeO_3_ sensor has the highest hysteresis.

#### 3.3.6. Stability Analysis

**Figure 23.**
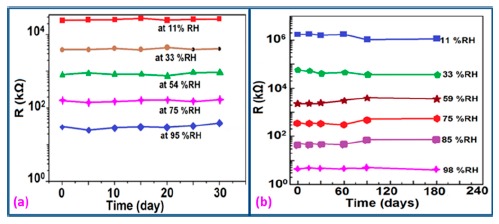
Stability response in terms of resistance or impedance *vs.* time characteristics for different iron-based sensor materials: (**a**) K-doped La_1−_*_x_*K*_x_*Co_0.3_Fe_0.7_O_3−δ_ perovskite; (**b**) mesoporous LaFeO_3_ sensors.

The stability of iron-based humidity sensors is observed from their resistance or impedance *vs.* time characteristics under different humidity conditions. In this instance, a K^+^-doped perovskite type iron-based humidity sensor (La_1−_*_x_*K*_x_*Co_0.3_Fe_0.7_O_3−δ_) showed very good stability for one month as shown in Figure 23a [108]. A very low change in impedance observed over time indicates the good long-time stability for practical use. In another work, the resistance variation with time for a mesoporous LaFeO_3_ sensor under different humidity conditions was observed for 6 months to analyze the long term stability as shown in Figure 23b [104] It has been observed that the impedance varies very slightly over the entire humidity region with time, which indicates that the mesoporous LaFeO_3_ humidity sensor has excellent long-term stability.

The stability of iron-based humidity sensors also can be examined from their humidity-resistivity characteristics. In contrast, Sn^4+^-doped MgFe_2_O_4_ humidity sensors shows very good stability at 20 °C, between 11% and 98% RH for up to 25 days as no significant changes were observed [105].

### 3.4. Silicon-Based Materials for Humidity Sensors

In order to achieve good performance, different materials such as ceramics, semiconductor oxides, carbon nanotubes, porous silicon, and organic polymers have been used for the development of humidity sensors. Among these materials silicon-based sensor materials have shown good responses for humidity measurement. Flexible surface properties, high temperature stability, resistance to aging and chemical assault have made silicon materials more robust and popular in the humidity sensor application field. To enhance the humidity sensing characteristics of silicon many researchers have developed different doping and coating techniques. Silicon-based humidity sensors can be fabricated with different silicon forms and/or dopants such as silica nanoparticle aerogels [110], mesoporous silica with different concentrations of Li^+^-ion doping [111], Li^+^-doped 3D periodic mesoporous silica [112], K^+^-doped 3D periodic mesoporous silica [113,114], mesoporous ZnO–SiO_2_ composites with various Si/Zn molar ratios [115], SiC nanowires grown on silicon nanoporous pillar arrays (Si-NPA) [116], and Zn_2_SiO_4_ thin film grown on silicon nanoporous pillar arrays (Zn_2_SiO_4_/Si-NPA) [117]. The most important materials and methods for silicon-based humidity sensors are listed in Table 2.

The electrical characteristics (resistance, capacitance, sensitivity, response time, hysteresis, stability) changes with the variation of frequency, temperature and relative humidity of silicon-based sensor materials are explained in the following subsections.

#### 3.4.1. Resistance/Impedance Variation with Relative Humidity

**Figure 24.**
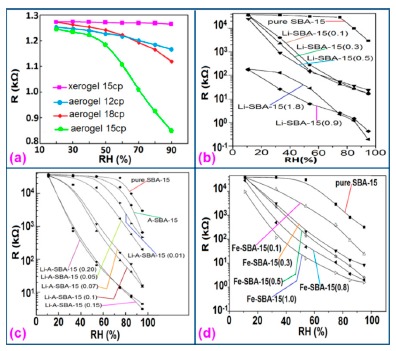
Impedance (or resistance) variation with relative humidity (RH) of silica-based humidity sensors for different materials: (**a**) xerogel or aerogel coatings; (**b**) different doping concentrations, Li^+^ in porous silica (SBA-15) [111]; (**c**) Li^+^ in synthetic porous silica (A-SBA-15) [118]; (**d**) Fe doped SBA-15 [119].

Silica aerogels and xerogels are attractive sensor materials and have been used as different coating materials in humidity sensors [110]. These authors have taken one xerogel sensor film coated at 15 cp, and three aerogel sensors film coated at 12, 15 and 18 cp for humidity sensing. The xerogel sensor showed poor sensitivity towards humidity and its impedance value remained independent at any RH value. On the other hand, the aerogel sensors showed better sensitivity than the xerogel sensor. The resistance value of the coating materials decreases more as the viscosity of the aerogel increases. To get the best results, the coating silica layer must have good uniformity as well as high porosity. At low viscosity the formation of a thin film of silica on the highly rough surface of the alumina substrate will not be perfect. However, at high viscosity the silica coating will be very thick and thus the impedance will be very high. It has also been noticed that layers of highly viscous solutions of silica on alumina substrates crack easily after supercritical drying, thus the electrical field conduction fails. Therefore, the silica aerogel sensor made at optimized viscosity, *i.e.*, 15 cp, shows the best linear sensing performance compared to the viscosities of 12 cp and 18 cp, as illustrated in Figure 24a [110].

In Figure 24a for an aerogel coating made at a viscosity of 15 cp, a dual response occurs due mainly to two consecutive water adsorption steps onto the silica surface [121]. In range of 20%–40% RH, the sluggish impedance change rate is related to the first adsorption stage, *i.e.*, surface interaction of hydrogen-bonded water molecules with isolated oxygen ions (≡Si−O−) in large silica mesopores under an electrostatic field [122]. Therefore, electrically activated protons are released from hydroxyl groups to form stable hydronium (H_3_O^+^) ions by interacting with adsorbing water (*i.e.*, H^+^ + H_2_O → H_3_O^+^). These stable charge carriers take part in the electrical conduction along with the water sub-monolayer during sensor operation [123]. In the second regime between 50% and 70% RH, the linear characteristic of impedance-RH curve is associated with the physisorption of bulk water on the silica aerogel film. The water layer formed by hydrogen bonding between water molecules acts as a continuous protonic conductor due to a greater conductivity of liquid water than that of air. Thus, a significant fast transport of charged H_3_O^+^ protons across the water band significantly improves the current density in the short path. The sensitivity of this aerogel is 900 Ω/% RH (*R*^2^ = 0.94) in the 20%–40% RH region and 9700 Ω/% RH (*R*^2^ = 0.99) in the 50%–70% RH region. At very high RH, the linear sensitivity may be lowered due to the bulk water that has physically filled up microporous voids of the silica film by capillary condensation in the presence of abundant moisture.

Doping agents in the different silicon particles can also significantly change the resistance, impedance or sensitivity of silicon-based humidity sensors. The doping of Li^+^ ions in mesoporous silica (e.g., SBA-15) greatly decreases the resistance by more than three orders of magnitude compared to undoped porous silica, SBA-15, and increases the humidity sensitivity with increased RH value in the 11%–95% RH range. The impedance-RH characteristics of undoped SBA-15 and Li-doped SBA-15 with different concentrations humidity sensors are illustrated in Figure 24b [111]. It has been found that the higher doping concentration is not at all suitable for humidity sensing applications. The resistance for pure SBA-15 shows low humidity sensitivity properties with the highest resistance value in the whole range of RH. Geng *et al.* showed that 10 wt % Li-doped SBA-15 exhibited the maximum humidity sensitive properties and best linearity. However, this same research group again synthesized mesoporous silica (A-SBA-15), which can be doped up to with 15 wt % Li^+^-ion having maximum sensitivity and best linearity (see Figure 24c) [118].

The addition of different doping materials in SBA-15 also changes its humidity characteristics drastically. Like Li ion, it has also been found that Fe doping decreases the impedance of SBA-15 with increase of RH considerably, compared to undoped SBA-15 (see Figure 24d) [119]. The pure SBA-15 sensor is sensitive to humidity above a RH value of 54%, however, Fe doped SBA-15 (Fe-SBA-15(*X*), where *X* is the weight ratio of Fe(NO_3_)_3_ to SBA-15, *X* = 0.1, 0.3, 0.5, 0.8, and 1.0) sensors showed humidity sensitivity in the whole RH range. Unlike Li^+^ doping, Fe^3+^-doped SBA-15 shows best performance at higher concentration (*i.e., X* = 0.5 ≈ 50 wt %) (see Figure 24d). Other silicon based materials such as SBA-16 [112], MCM-41 [120], and SiO_2_ [115] matrix have been used in humidity sensing applications. Mesoporous silica has also been doped or composited with other materials such as K_2_CO_3_ [113], ZnO [115], and MgO [124] to improve the conductivity by reducing the impedance or resistance in humidity sensors.

#### 3.4.2. Effect of Frequency on Resistance-RH Characteristics

**Figure 25.**
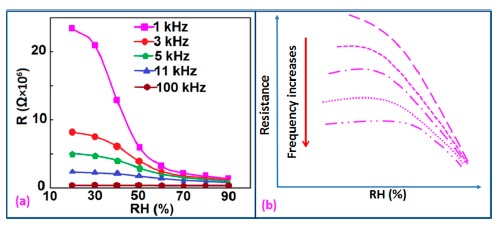
Resistance variation with relative humidity at different frequencies for different silicon-based materials: (**a**) silica aerogel coated at a viscosity of 15 cp; (**b**) for doped silicon-based sensor materials.

Frequency has a great impact on the resistance or impedance variation with relative humidity and doping elements in silicon-based sensor materials. It has been found that in a low humidity regime the resistance of silica aerogel film decreases remarkably with increasing frequency. The resistance difference between two working frequencies becomes progressively smaller with increasing RH value. It has also been observed that at higher frequencies, *i.e.*, more than 100 kHz, the resistance becomes independent over the whole RH range. The resistance responses of a silica aerogel-coated sensor (viscosity of 15 cp) at different frequencies as a function of relative humidity at 25 °C are shown in Figure 25a [110].

Like silica coating materials (e.g., xerogels or aerogels), the impedance of doped silica sensors decreases greatly with increasing relative humidity at a lower frequency range, but at higher frequency the rate of impedance change becomes lesser with increasing % RH. For doped silicon-based sensors, the dopants significantly enhance the linearity of the sensors besides decreasing the impedance or resistance value. A schematic representation of impedance *vs.* RH at different frequencies is illustrated in Figure 25b.

#### 3.4.3. Effect of Frequency on Capacitance-RH Characteristics

**Figure 26.**
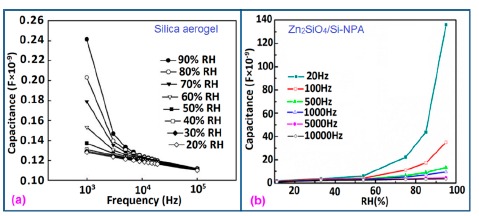
Capacitance as a function of frequency at different RH values for different silicon-based materials. (**a**) Silica aerogel coated as 15 cp viscosity sensor material [110]; (**b**) Zn_2_SiO_4_/Si-NPA humidity sensor [117].

The frequency has also significant role in the variation of capacitance of silicon-based sensor materials. At a low frequency range, the capacitance value is generally high and it increases with increased RH value. However at higher frequency (*i.e.*, greater than ≥5 kHz), the change in capacitance with the RH becomes negligible. The capacitance as a function of frequency at different RH values for silica aerogels coated on a 15 cp viscosity sensor material is depicted in Figure 26a [110] and for a Zn_2_SiO_4_/Si-NPA humidity sensor as illustrated in Figure 26b [117]. For silica aerogels, the curves become nonlinear at 40% RH and higher. A similar observation was made for SiC nanowires grown on silicon nanoporous pillars (nw-SiC/Si-NPA) [116].

#### 3.4.4. Response and Recovery Time Analysis

For a good sensor, the response and recovery time of humidity sensors must be very small. These may be different for different materials, doping ions, conditions and procedures and thus the RH *vs.* time plots for different sensors change accordingly. The response and recovery time of some different silicon-based humidity sensor materials are listed in Table 3.

**Figure 27.**
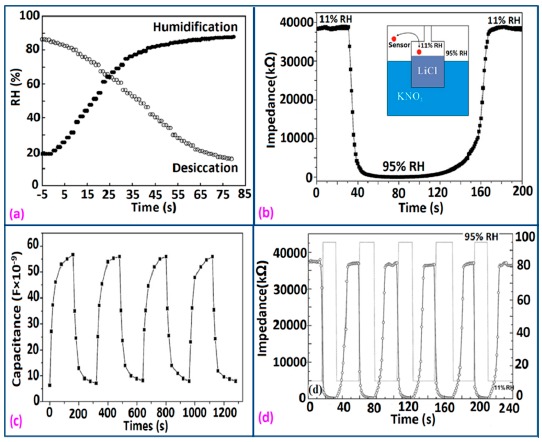
Response and recovery times of different silicon based materials: (**a**) high response (during humidification) and recovery (during desiccation) times showed by a 15 cp silica aerogel sensor at 25 °C and 1 kHz [110]; (**b**) low response but high recovery time shown by Fe-SBA-15(0.5) from 11% to 95% RH measured at 100 Hz; the inset represents a schematic f response measuring equipment [119]; (**c**) high response and recovery time shown by nw-SiC/Si-NPA in between 11% and 95% RH at a constant frequency of 100 Hz [116]; (**d**) very low response and recovery times shown by a MgO-SBA-15(*R* = 1) sample [124].

The response and recovery time of some selected silicon-based humidity sensor materials are depicted in Figure 27a–d to compare the response and recovery times shown by the different materials. From these results it is observed that Zn_2_SiO_4_/Si-NPA is the only sensor whose recovery time is more than the response time, but all the remaining sensors have greater recovery times than response times. This effect occurs due to the specific surface morphology of Zn_2_SiO_4_/Si-NPA. It is observed that all the Zn_2_SiO_4_-covered pillars are well separated and form a regular array [117]. The valleys around the pillars connect to each other and form a well-defined channel network. This would provide an effective pathway for the transport of the vapor in and out of the pillar layer, which would surely shorten response time. In addition, the MgO-SBA-15(*R* = 1) sensor and K^+^ ion doped K-SBA-15(0.5) sensors have shown lower response times, whereas a Zn_2_SiO_4_/Si-NPA sensor has shown the smallest recovery time.

#### 3.4.5. Hysteresis Characteristics Analysis

**Figure 28.**
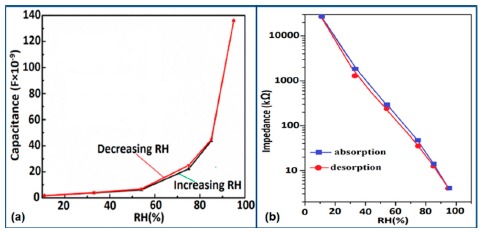
Hysteresis response of different silicon based materials. (**a**) Hysteresis in capacitance *vs.* % RH of Zn_2_SiO_4_/Si-NPA [117]; (**b**) Schematic representation of hysteresis in impedance *vs.* % RH of MgO-SBA-15(*R* = 1) sample.

For a perfect based humidity sensor, the hysteresis value must be very small. The hysteresis values of different silicon-based humidity sensors are listed in Table 3. The hysteresis responses of some selected silicon-based sensors are depicted in Figure 28. Hysteresis in capacitance *vs.* % RH of Zn_2_SiO_4_/Si-NPA is depicted in Figure 28a [117], while the hysteresis of a MgO-SBA-15(*R* = 1) sample is illustrated in Figure 28b by its impedance *vs.* % RH plot.

From the above comparison it can be seen that the MgO-SBA-15(*R* = 1), Zn_2_SiO_4_/Si-NPA sensor and ZnO−SiO_2_(*R* = 1) sensor materials have the lowest hysteresis and the Li-SBA-15(0.1) sensor has the highest hysteresis.

#### 3.4.6. Stability Analysis

**Figure 29.**
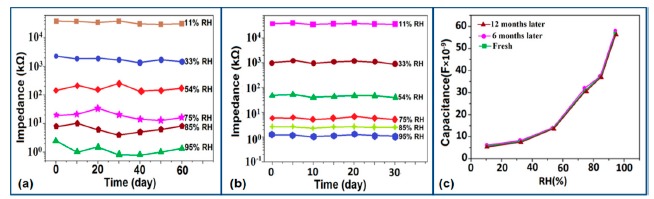
Stability response of different silicon-based humidity sensor materials. (**a**) Change in impedance with increasing time for Fe-SBA-15(0.5) measured at a constant frequency of 100 Hz and at different % RH; (**b**) change in impedance with increasing time for the sensor based on Li doped silicon, *i.e.*, SBA-16/Li^+^(0.1), measured at a constant frequency of 100 Hz and at % RH; (**c**) variation of capacitance with increasing % RH for the sensor based on nw-SiC/Si-NPA material at different experimental time intervals.

The coating of different silica gels is also suitable for humidity sensors sine it exhibit good stability and reproducibility over time. The iron doped mesoporous silica (*i.e.*, Fe-SBA-15(0.5) or Li-doped SBA-16) sensors has shown acceptable change in the impedances, proving the good stability as shown in schematic Figure 29a,b.

Similarly, the stability of nw-SiC/Si-NPA sensors was evaluated by comparing the capacitance–RH curves measured at 100 Hz and they show excellent stability as the capacitance value after a long time (6-month and 12-month, storage) remained almost unchanged (see Figure 29c).

### 3.5. Polymer-Based Materials for Humidity Sensors

The popularity of polymer-based sensors is increasing day by day due to their high sensitivity, fast response, easy preparation, and low cost. However, instability in humid environments is the main hindrance in their wider application and further development. To overcome this problem, several methods such as introduction of hydrophobic groups by copolymerization and grafting, crosslinking, application of protective films, interpenetration of network structures, or formation of organic/inorganic hybrids have been proposed so far. In contrast, thermoset polymers have shown excellent humidity sensing properties. Cross-linked thermoset polymer network structures are a primary criterion for a humidity sensor. Different copolymers may be coated on different ceramic substrates or can be used directly as bulk the phase. Polymeric sensors have mainly been used in capacitive type and resistive type humidity sensors. Polymer electrolytes or polymer–salt complexes are used for the fabrication of resistive type sensors, whereas hydrophobic polymers are used for capacitive-type sensors. Different types of polymeric humidity sensors are listed chronologically in Table 4. The main advantages of the polymeric sensors are excellent flexibility, outstanding purity and very high condensation resistance. The hysteresis loss of polymeric humidity sensors is very low (nearly 1% RH) in comparison to other ceramic sensors. The key factors and characteristics of the polymeric humidity sensors are also discussed in the following subsections. The variation of the electrical characteristics (resistance, capacitance, sensitivity, response time, hysteresis, stability) with the variation of frequency, temperature and relative humidity of polymer-based sensor materials are explained in the following subsections.

#### 3.5.1. Resistance or Impedance Variation with Relative Humidity

**Figure 30.**
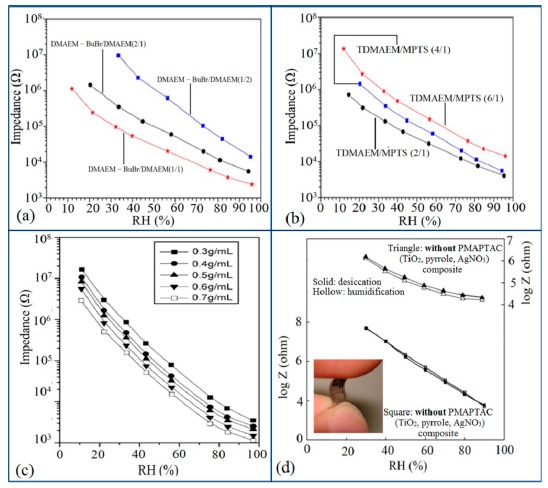
Resistance or impedance variation with relative humidity at different doping/coating concentrations: (**a**) Si-PE copolymer crosslinked with DBB and different molar ratios of TDMAEM/MPTS = 4/1 and DMAEM—BuBr/DMAEM [125]; (**b**) Si-PE copolymer crosslinked with DBB at different molar ratios of DMAEM-BuBr/DMAEM = 1/1 and TDMAEM/MPTS [125]; (**c**) for different ratios of 3-aminopropyltriethoxysilane (APTS) and quaternized *n*-butyl bromide (BB), the impedance decreases slightly and it is very high in the low humidity region [128]; (**d**) the change in impedance of a PMAPTAC-induced composite (TiO_2_ = 0.048 g, pyrrole = 0.125 g, AgNO_3_ = 0.0314 g, PMAPTAC = 0.08 g)-based sensor [129] decreases greatly in comparison to the best composite (TiO_2_ = 0.0012 g, pyrrole = 0.125 g, AgNO_3_ = 0.0314 g) sensor reported by Su and Huang [129]. Inset: A flexible sensor based on a TiO_2_NPs/PPy/PMAPTAC composite thin film while it was bent and its impedance-RH characteristics [130].

In general, the resistance or impedance level of polymeric humidity sensors is significantly higher than that of ceramic- or silicon-based humidity sensors. For different copolymers and different doping or coating substrate materials, the resistance or impedance value of the humidity sensors changes significantly. It has been seen that the impedance of the polyelectrolyte (PE) copolymer crosslinked with dibromobutane (DBB) does not increase gradually with an increase of dimethylaminoethyl methacrylate (DMAEM) content, which provides crosslinking and quaternizing points in the copolymer (see Figure 30a) [125]. On the other hand, the impedance decreased abnormally as the content of hydrophobic γ-methacryloxypropyltrimethoxysilane (MPTS) co-monomer increased in the polyelectrolytes (see Figure 30b) [125]. An optimized molar ratio between TDMAEM (DMAEM—BuBr + DMAEM) and MPTS of 2 with a mixture of DMAEM—BuBr and DMAEM at molar ratio of 2 has exhibited the best humidity sensitive characteristics. In contrast, MPTS can enhance the flexibility and adhesion of humid membranes to the electrode substrate [126], whereas DMAEM-BuBr contains quaternary nitrogens, which can act as ion moieties in Si-PE molecules, and has potential for enhancing the sensor performance [127].

In another study, it has also been observed that due to using of different ratios of different polymers such as 3-aminopropyltriethoxysilane (APTS) which is quaternized with n-butyl bromide (BB), the impedance decreases slightly and gives very high impedance value at low humidity region (see Figure 30c) [128]. Polymeric nanocomposites have also been used as successful potential candidates for humidity sensors. In a composite of TiO_2_ nanoparticles, polypyrrole and AgNO_3_ based humidity sensor, impedance varies significantly with RH with addition of different doping concentration. The impedance decreases with increases of doping concentration in all relative humidity range. The main advantages of the conducting polypyrrole polymers (PPy) are relatively good environmental stability and most importantly its surface charges can easily be modified by different dopants by easy synthesis method [129]. It has also been noticed that the addition of PMAPTAC in a polymer electrolyte enhances the sensitivity characteristics of composite (TiO_2_, pyrrole, AgNO_3_) humidity sensor [130]. The basic group, −N^+^(CH_3_)_3_Cl^−^ present in MAPTAC polymer electrolyte is responsible for improving the humidity sensitivity and also makes it flexible to exhibit favorable electrical performance when they were bent as shown in the inset of Figure 30d [130]. The impedance of PMAPTAC induced composite (TiO_2_ = 0.048 g, pyrrole = 0.125 g, AgNO_3_ = 0.0314 g, and PMAPTAC = 0.08 g) based sensor decreases greatly in comparison to the best composite (TiO_2_ = 0.0012 g, pyrrole = 0.125 g, and AgNO_3_ = 0.0314 g) sensor reported by Su and Huang, 2007 [129]. It has been observed that the composite with PMAPTAC [130] gives far better linearity than that of without PMAPTAC composite [129] sensor.

High impedance at low humidity is a general trend for many copolymer films. The impedance of quaternizedpolypyrrole (PPy) composite film shows a change from 7.8 × 10^5^ to 7.0 × 10^4^ Ω with an increase in RH from 11% to 95% with greater linearity and good sensitivity (0.0370), but copolymer film has poor linearity and less sensitivity (0.0305) [133]. For the doping polymers, it has been noticed that due to addition of dopant (e.g., Fe^2+^-ion) in polymer (e.g., polypyrrole) its sensitivity increases and impedance decreases sharply with better linearity as increase of RH value [134]. Almost similar trend has been noticed in gold nanoparticles (AuNPs) reinforced generation 1 amine terminated polyamidoamine (PAMAM) dendrimer (G1-NH_2_) polymer [135]. For G1-NH_2_ polymer sensor, almost no impedance change occurred from 30% RH up to 70% RH, the impedance falls gradually in the range of 70%–90% RH [135]. The impedance, however, of AUNPs reinforced polymer (G1-NH_2_-AuNPs) film sensor, was markedly reduced over a wide range of RH, i.e., 30%–90% RH [135]. On the other hand, the impedance of a copolymer also can be tuned by changing of coupling agent. The impedance of a copolymer decreases as the amount of N-(3-dimethylaminopropyl)-N'-ethylcarbodiimide hydrochloride (EDC) increases, because increasing of EDC content increases the degree of anchoring of methyl methacrylate (MMA) and [3-(methacrylamino)propyl] trimethyl ammonium chloride (MAPTAC) copolymer (poly-MMA-MAPTAC) onto the 3-mercaptopropionic acid/gold (MPA/Au) surface by the formation of peptide bonds [136]. The EDC acted as a peptide coupling reagent in this copolymer sensor.

#### 3.5.2. Effect of Frequency on Resistance-RH Characteristics

**Figure 31.**
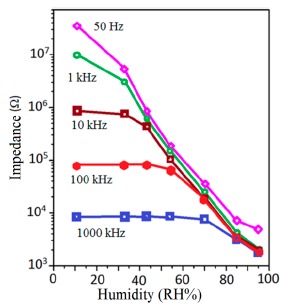
A schematic representation of impedance variation with relative humidity of a TiO_2_ and polystyrene sulfonic acid sodium (NaPSS) composite sensor at different frequencies.

The frequency also significantly affects the impedance of polymeric humidity sensor materials. At low RH, the frequency influences the impedance more significantly than in the case at high RH. The addition of PMAPTAC to a TiO_2_/polypyrrole sensor enhances its frequency effect on impedance [129]. The similar observation is also shown for polymers based on other polymeric materials such as flexible PMAPTAC/TiO_2_/polypyrrole [130], TiO_2_/ polystyrene sulfonic acid sodium (NaPSS) [132], Fe^2+^ doped polypyrrole [134], G1-NH_2_-AuNPs film [135], poly-MMA-MAPTAC anchored MPA/Au [136], and so on. The frequency at which the sensors show the best linearity is different for different polymeric materials. The range of RH values is also different for different polymeric humidity sensors at the same frequency. Sun *et al.* measured the impedance of a TiO_2_ and polystyrene sulfonic acid sodium (NaPSS) composite sensor over a wide range of frequencies between 50 Hz and 10^6^ Hz, as shown in Figure 31 [132]. They observed that at low RH, the frequency affected the impedance more strongly and linearly than at high RH. When the frequency was high, the electrical field direction was changed so rapidly that the polarization of the adsorbed water cannot keep up with this rapid change, and as a result the dielectric constant remained small and independent of RH (see the curve for 1000 kHz in Figure 31).

#### 3.5.3. Effect of Temperature on Resistance-RH Characteristics

**Figure 32.**
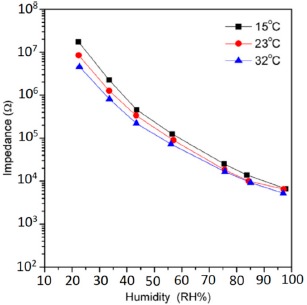
Impedance variation with relative humidity of a 2-(dimethylamino)ethyl methacrylate (DMAEMA), *n*-butyl bromide (BB)-based composite humidity sensor at different temperatures [131].

Temperature is another important key parameter which can change the behavior of resistance or impedance *vs.* relative humidity curves significantly for polymer based sensors. As the temperature increases, the RH characteristic curve shifts toward lower impedance values. For example, a 2-(dimethyl-amino)ethyl methacrylate (DMAEMA), *n*-butyl bromide (BB)-based composite humidity sensor shows impedance variation with temperature over the whole humidity range and this is clearly illustrated in Figure 32 [131]. The temperature coefficient is estimated to change from −0.36% to −0.42% RH/°C between 15 and 32 °C, and the temperature dependence tendency is found to decrease with increasing humidity. An almost similar trend in the effect of temperature on resistance or impedance *vs.* significant relative humidity characteristics has been observed for polymer-based sensor materials such as TiO_2_/pyrrole/AgNO_3_ composite [129], TiO_2_/PMAPTAC composite [130], G1-NH_2_-AuNPs film [135], poly-MMA-MAPTAC anchored onto MPA/Au surface with 200 mM EDC [136], and so on.

#### 3.5.4. Response and Recovery Time Analysis

**Figure 33.**
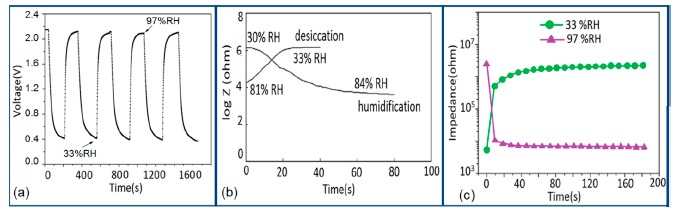
Response and recovery time behavior of different polymer-based materials. (**a**) APTS and BB [128]; (**b**) TiO_2_/polypyrrole [129]; (**c**) DMAEMA and BB.

Response and recovery times are also significant features for estimation of polymeric humidity sensors. Response and recovery times of different polymeric humidity sensors are listed in Table 5. The response or recovery time of polymeric humidity sensors also can be measured by their voltage *vs.* RH characteristics as illustrated for APTS and BB one [128] in Figure 33a. A comparison of response or recovery times of three other different types of polymer- or copolymer-based humidity sensor materials, such as TiO_2_/polypyrrole [129], and DMAEMA and BB [131], measured from impedance *vs.* % RH plots are shown in Figure 33b,c, respectively.

#### 3.5.5. Hysteresis Characteristics Analysis

**Figure 34.**
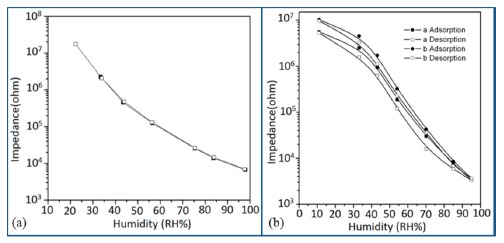
Hysteresis response of different polymer based materials. (**a**) DMAEMA and BB based humidity sensor [131]; (**b**) TiO_2_/NaPSS composite [132].

Hysteresis properties of different polymeric humidity sensors are listed in Table 5. Polymers containing a hydrophobic monomer (MPTS) show high hysteresis and large reversible responses, but the Si-PE copolymer cross-linked with DBB in a DMAEM-BuBr/DMAEM molar ratio of 4 shows very small hysteresis (∼2% RH) [125]. A very small hysteresis of only ∼1% RH is also shown for the DMAEMA and BB-based humidity sensor as illustrated in Figure 34a [131].

A very small hysteresis loop was observed in a TiO_2_/NaPSS composite compared to the pure polymer NaPSS humidity sensor as shown in Figure 34b [132]. Another composite quaternized polypyrrole polymer film based humidity sensor shows a relatively wide hysteresis loop, which indicates that the rate of the desorption process of the adsorbed water is slower than that of the water adsorption process [132].

#### 3.5.6. Stability Analysis

**Figure 35.**
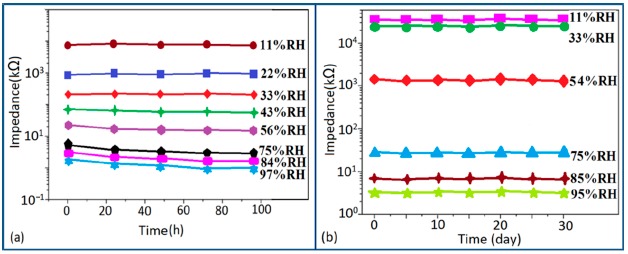
Long-term stability response of silicon-based humidity sensors. (**a**) based on APTS and BB, after operating at 93% RH and 38 °C with applied 0.2 V at 1 kHz, measured at different relative humidities (concentration of precursor solution: 0.5 g/mL); (**b**) Fe^2+^-doped polypyrrole up to 30 days at different RH values.

The stability of polymer-based humidity sensors materials is excellent in terms of long term applications. In order to analyze the stability of an APTS and BB-based humidity sensor, Lv *et al.* varied the impedance and observed that the sensor variation was changed little over almost the entire humidity range (11%–97%) after 96 h, showing its excellent stability (Figure 35a) [128].

In this context, Fe^2+^-doped polypyrrole sensors have shown excellent stability up to 30 days at different RH values as illustrated in Figure 35b [134]. The stability response of other polymer-based humidity sensors, such as TiO_2_/polypyrrole [129], G1-NH_2_-AuNPs film [135], and poly-MMA-MAPTAC anchored onto MPA/Au surface with addition of 200 mM EDC [136], have also been measured as very good, with extremely low variation of impedance or resistance for long term applications.

### 3.6. Tin-Based Materials for Humidity Sensors

Metal oxide semiconductors such as tin oxide (SnO_2_), zinc oxide (ZnO), tungsten oxide (WO_3_) and iron oxide (Fe_2_O_3_) are the most popular humidity sensing materials. The fundamental principle of all these metal oxide semiconductors is based on electrical conductivity, which changes with the composition of the surrounding gas atmosphere. Recently, most of the development work has been focused on SnO_2_-based materials, such as K^+^-doped SnO_2_−LiZnVO_4_ [137], KCl-doped SnO_2_ nanofibers [1], and SnO_2_−LiZnVO_4_ ceramic [138], La^3+^ and K^+^ co-doped Ti_0.9_Sn_0.1_O_2_ thin films [139], SnO_2_ nanoparticles [140], KCl-doped nanoporous Ti_0.9_Sn_0.1_O_2_ thin films [141], and ZnSnO_3_ cubic crystallites [142], due to their large surface areas, high surface activity, good gas sensitivity, lower working temperature and adaptability to sense different gases with the addition of suitable dopants. Different types of tin oxide-based humidity sensors and their synthesis methods are listed in Table 6. The electrical characteristics (resistance, capacitance, sensitivity, response time, hysteresis, stability) variation with the variation of frequency, temperature and relative humidity of tin-based sensor materials are explained in the following subsections.

#### 3.6.1. Resistance or Impedance Variation with Relative Humidity

**Figure 36.**
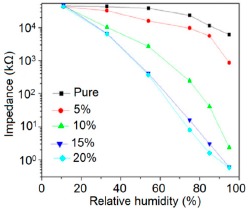
Impedance variation with relative humidity of KCl-doped SnO_2_ nanofibers at doping concentrations of 5%, 10%, 15%, 20% KCl [1].

Doping concentration has a great impact on the impedance variation of tin oxide-based composites. From the experimental analysis it is confirmed that the impedance of the composite decreases with increasing doping concentration as distinctly illustrated in Figure 36 for KCl-doped SnO_2_ nanofiber sensors at doping concentrations of 5%, 10%, 15%, 20% KCl [1]. The 15% KCl in SnO_2_ sensor shows the best linearity and good sensitivity [1].

For other materials such as a K^+^-doped SnO_2_−LiZnVO_4_ humidity sensor [137], or LiZnVO_4_ added in SnO_2_ [138], and so on have shown similar kind of effects. In all these systems, the pure humidity sensor shows poor sensitivity in the whole range of humidity values, but as doping concentration is increased, the sensor impedance decreases greatly, sensitivity is enhanced, and the linearity deteriorates at high RH.

#### 3.6.2. Effect of Frequency on Resistance-RH Characteristics

**Figure 37.**
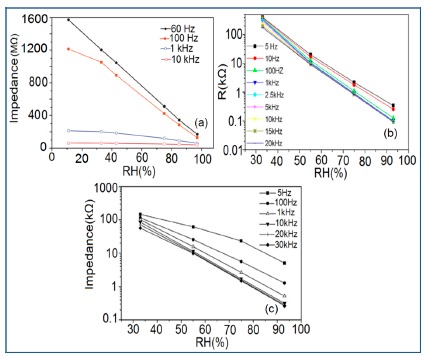
Resistance or impedance variation with relative humidity at different frequency for different materials: (**a**) ZnSnO_3_ cubic crystallite film humidity sensor [142]; (**b**) KNO_3_ doped SnO_2_–LiZnVO_4_ [137]; (**c**) LiZnVO_4_ doped SnO_2_ [138].

The different tin oxide-based humidity sensors show their best impedance-RH characteristics at different operating frequencies. A significant nonlinearity is observed in the low humidity range at high operating frequency and good linearity is found in the high humidity region as well as at low operating frequency. It has been seen that impedance decreases with increasing frequency for any RH, but the best linearity of the impedance *vs.* RH curve is observed at lower frequency levels, for example, the best linearity appeared at 100 Hz for KCl-doped SnO_2_ nanofibers [1] and 60 Hz for ZnSnO_3_ cubic crystallite film humidity sensors [142], as shown in Figure 37a.

The impedance became independent of the humidity at a frequency range nearly greater than 1 kHz. This is because at high frequency, the electrical field direction changes so fast that the polarization of the water cannot catch up with it, and as a result the dielectric constant becomes small and independent of RH. Conversely, the direction of electrical field changes slowly at low frequency and subsequently a high space-charge polarization appears on the adsorbed water, and thus impedance or resistance changes significantly at low frequency. In contrast, a few materials such as KNO_3_ doped SnO_2_−LiZnVO_4_ [137] and LiZnVO_4_ doped SnO_2_ [138] humidity sensors show excellent linearity at any frequency over the whole humidity range, as depicted in Figure 37b,c, respectively.

#### 3.6.3. Response and Recovery Time Analysis

The response and recovery time of some tin oxide-based humidity sensors such as KNO_3_-doped SnO_2_−LiZnVO_4_ [137], KCl-doped SnO_2_ nanofibers [1], LiZnVO_4_-doped SnO_2_ [138], KCl-doped nanoporous Ti_0.9_Sn_0.1_O_2_ thin films [141], and ZnSnO_3_ cubic crystallite [142], are listed in Table 6. From the reported data it is clear that the KCl-doped SnO_2_ nanofiber humidity sensor has the smallest response and recovery time.

#### 3.6.4. Hysteresis Characteristics Analysis

**Figure 38.**
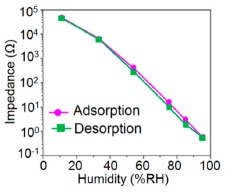
Hysteresis response of a KCl-doped SnO_2_ nanofiber humidity sensor.

All the tin oxide-based humidity sensors show very narrow hysteresis loops of less than 6%. So far the KCl-doped SnO_2_ nanofiber humidity sensor has shown smallest hysteresis of 3% among the tin oxide-based humidity sensors, as seen in Figure 38 [1].

#### 3.6.5. Stability Analysis

At low RH, the stability is very high for all the tin oxide-based humidity sensors as very little change in impedance or resistance is observed at low RH conditions even up to 25 months of study. Considering all the above tin oxide-based sensors, the LiZnVO_4_-doped SnO_2_ sensor impedance variation was almost constant, hence it shows the best stability compared to other sensors.

### 3.7. Titanium-Based Materials for Humidity Sensors

Titanium oxide (TiO_2_) is a most popular metal oxide semiconductor material which has enormous applications in environmental cleaning and protection, solar cells, photocatalysis, and chemical sensors. Anatase (tetragonal), rutile (tetragonal), and brookite (orthorhombic) are the three most available crystallographic structures of TiO_2_ crystal. The catalytic performance, photocatalytic activities, as well as sensing properties of TiO_2_-based devices are influenced by the differences in crystallographic structures. The devices were fabricated by evaporating metal contacts on a SiO_2_ layer thermally grown on a silicon substrate. The humidity sensing characteristics of CdTiO_3_ nanofibers prepared by the electrospinning method were analyzed by Imran *et al.* [143]. Bi_0.5_(Na_0.85_K_0.15_)_0.5_Ti_0.97_Zr_0.03_O_3_ (BNKTZ) sensing materials were successfully synthesized via a simple metal–organic decomposition method by Wang *et al.* [144] and their humidity sensing properties, which are listed in Table 7, were successfully analyzed. The variation of electrical characteristics (resistance, capacitance, sensitivity, response time, hysteresis, stability) with the variation of frequency, temperature and relative humidity of titanium-based sensor materials are explained in the following subsections.

#### 3.7.1. Resistance or Impedance Variation with Relative Humidity

**Figure 39.**
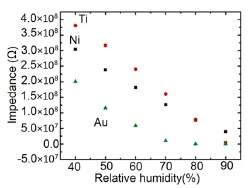
Impedance variation with relative humidity of TiO_2_-based humidity sensors with different metallic doping electrodes [143].

Like other humidity sensors, the resistance or impedance of titanium oxide-based humidity sensors also decreases with increasing RH. The effect of different metallic electrodes (e.g., Ti, Ni, and Au) on the humidity sensing properties of electrospun TiO_2_ nanofibers is different, as shown in Figure 39 [143].

The impedance of three TiO_2_ nanofibers sensors with different metallic electrodes such as Ti, Ni, and Au becomes smaller as the RH increases from 40% to 90% RH at room temperature. The size of the impedance range decrease is quite different for different electrode materials, and it is 300–38 MU for Ni-electrode sensor, 381–4.7 MU for Ti-electrode sensor and 199–0.03 MU for a Au-electrode sensor in the humidity range from 40% to 90% RH. Ti-electrode and Ni-electrode sensors show quite linear behavior in this range, however the Au-electrode sensor offers low impedance with significantly poorer linear behavior as compared to the other two metallic electrode sensors. The Ti-electrode has shown noticeably higher sensitivity of (7.53 MU/%RH) compared to Ni-(5.29 MU/%RH) and Au-(4.01 MU/%RH) electrode sensors at 100 Hz.

#### 3.7.2. Effect of Frequency on Resistance-RH Characteristics

The impedance or resistance of titanium oxide-based humidity sensors decreases as RH increases with operating frequency and it is influenced significantly by the frequency in different humidity ranges. Maximum linearity but high resistance or impedance is observed at lower operating frequency in the whole RH range for all these humidity sensors. However, at high operating frequency the best linearity is observed only at higher RH range and the impedance or resistance becomes independent of the RH at lower frequencies. The observed flat or independent impedance curves are attributed to dielectric phenomena. At higher frequency, the adsorbed water molecules are difficult to polarize, which leads to an insignificant decrease in impedance. Most of these materials show their best impedance-RH curve linearity at nearly 100 Hz.

**Figure 40.**
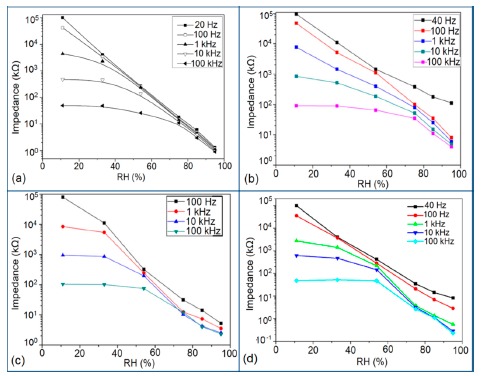
Different linearity in impedance variations with RH at different frequencies for different sensor materials: (**a**) KCl-doped TiO_2_ nanofibers calcined at 600 °C [146]; (**b**) BKT [147]; (**c**) BNT–BKT [150]; (**d**) BNKTZ [144] humidity sensors.

The impedance variations with RH at different frequencies are depicted in Figure 40a [146] for KCl-doped TiO_2_ nanofibers calcinated at 600 °C, in Figure 40b [147] for a BKT humidity sensor, Figure 40c [150] for a BNT–BKT sensor, and in Figure 40d [144] for a BNKTZ humidity sensor. It has been seen that the impedance of the BKT sensor is influenced especially at low RH, and the impedance decreases remarkably with an increase of the frequency. The high humidity sensitivity and the good linearity of the impedance *vs.* RH curve appear in the low frequency region, and the best linearity appears at 100 Hz. The BNT–BKT sensor impedance decreases remarkably with increasing frequency at low RH, and the impedance difference between two adjacent curves becomes progressively smaller with increasing RH. A similar kind of behavior was also found in the other nanomaterials such as CdTiO_3_ nanofiber [143], BaTiO_3_ nanofiber [149], and Na_2_Ti_3_O_7_ nanowire sensors [145].

#### 3.7.3. Effect of Temperature on Resistance-RH Characteristics

Temperature is another important key parameter for humidity sensors because humidity always depends on the ambient temperature. The impedance decreases with increasing ambient temperature since the applied thermal energy activates the mobile charge carriers within a stable water layer.

**Figure 41.**
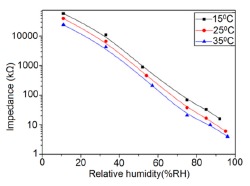
Impedance variation with relative humidity of Na_2_Ti_3_O_7_ nanowires sensor at different temperatures [145].

The impedance *vs.* RH plots of Na_2_Ti_3_O_7_ nanowires sensor at different temperatures measured at 100 Hz is shown in Figure 41 [145]. The average temperature coefficient between 15 and 35 °C is about −0.2% RH/°C in the humidity range of 11%–95% RH.

#### 3.7.4. Effect of Frequency on Capacitance-RH Characteristics

**Figure 42.**
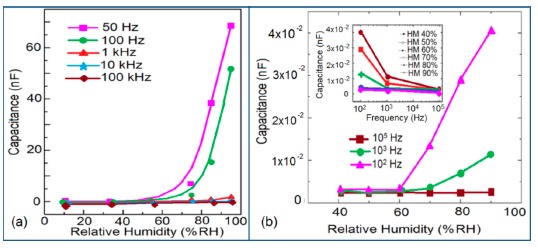
Capacitance variation with relative humidity at different frequencies for different nanomaterials: (**a**) BaTiO_3_ nanofiber; (**b**) TiO_2_ nanofiber.

It has been noticed that the capacitance increases with an increase in RH at low frequencies from 50 to 100 Hz. However, at high frequencies, greater than 1 kHz, the capacitance values become very low at any RH and are almost independent of the RH. The relationship between capacitance and RH of BaTiO_3_ nanofiber sensor at different frequencies from 50 Hz to 100 kHz is depicted in Figure 42a [149]. A relation between capacitance and relative humidity at different frequencies for TiO_2_ nanofiber is Figure 42b and the inset of Figure 42b depicts the variation of capacitance with frequency under different absolute humidity conditions [143]. It can be noticed that the capacitance increases gradually as the RH increases, but it changes rapidly by lowering the frequency. At high RH values, the capacitance increases as the frequency decreases. At higher frequency, *i.e.*, >1 kHz, the capacitance becomes very small and hardly changes with humidity.

#### 3.7.5. Response and Recovery Time Analysis

Response and recovery times of different titanium oxide-based humidity sensors are listed in Table 7. From the above data it is observed that the KCl-doped TiO_2_ nanofiber humidity sensor has the lowest response and recovery time.

#### 3.7.6. Hysteresis Characteristics Analysis

Titanium-based humidity sensors in general have lower hysteresis and the values are illustrated in Table 7. It is evident that the Ti-electrode sensor has lower hysteresis values as compared with those for the Ni-electrode and Au-electrode sensors [153]. The ZnO/TiO_2_core/shell nanorod sensor has high hysteresis, but has very good repeatability [152]. From the above results it is clear that the BKT humidity sensor has lowest hysteresis value [147].

#### 3.7.7. Stability Analysis

**Figure 43.**
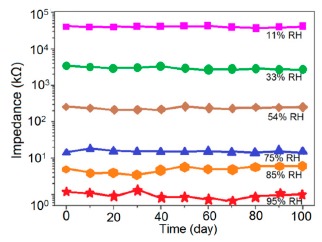
Stability response of a KCl-doped TiO_2_ nanofiber humidity sensor.

The stability of titanium oxide-based humidity sensors is high at low frequency but they have high impedance values, even after exposure for up to 100 days. Considering various titanium-based materials, it has been noticed that the titanium oxide-based humidity sensors show very good stability in terms of insignificant change in impedance with increasing time up to 75% RH, and beyond that RH, the resistance or impedance varies significantly. It has also been reported that the KCl-doped TiO_2_ nanofibers show better best linearity and the quickest response compared with pure anatase or rutile structures [146]. In order to explain the stability of the best titanium oxide-based humidity sensor, we select a KCl-doped TiO_2_ nanofiber humidity sensor, which was exposed to air for 100 days to measure its impedances at various RH levels. As shown in Figure 43, there is almost no change in the impedance up to 75% RH [146]. This confirms directly the prominent stability of this sensor. Like any other sensor material, the titanium oxide-based humidity sensor materials also forms water layers with increasing RH on the surface of nanofibers by a physisorption mechanism. The mechanism of the dissociation of the doping material has an important role in a material in order to show the best performance or stability. Normally, in ceramic and porous materials, the water-related conduction occurs mainly through surface mechanisms [154]. However, for films, a large increase in electrical conductivity with increasing RH of TiO_2_ nanofibers may be related to the adsorption of water molecules on the surface of the sensing film. For nanofibers, a high local charge density and a strong electrostatic field are provided by the tips and defects of the nanofibers, and this can promote the dissociation of water and ionic salt molecules [155]. In case of the KCl-doped TiO_2_ nanofiber humidity sensor, the dissociation of KCl supplies protons and ions (such as H^+^, H_3_O^+^, K^+^, Cl^−^) as charge carriers for the hopping transport. In this system, H_3_O^+^ may be hydrated in the presence of sufficient adsorbed water since hydration of H_3_O^+^ is energetically favored in liquid water [123,146,156]. Although the initial and final states are the same (water molecule and hydronium ion) according to the ion transfer mechanism [157], the transfer of hydronium (H_3_O^+^) is quite easy as the energy is equivalent. Simultaneously, KCl dissolves in the adsorbed water and plays a major role in carrier conduction by dissociating into K^+^ and Cl^−^ ions. However, when the RH is increased further, more of the ionic salt (*i.e.*, KCl) dissociates into free ions (*i.e.*, K^+^ and Cl^−^). This enhances the transfer process of the KCl-doped TiO_2_ nanofibers, and as a result, the linearity of the impedance *vs.* RH curve improves significantly by doping KCl in the whole range of 11%–95% [146].

Qi *et al.* [146] had also speculated that the crystallographic structure may change the specific surface area (*i.e.*, surface-to-volume ratio) of their KCl-doped TiO_2_ nanofibers, but all their samples exhibited relatively low specific surface areas. Hence, eventually, they considered dissociation phenomena along with crystallographic structure in order to explain the performance or linearity of TiO_2_- and KCl-doped TiO_2_ nanofiber sensors. According the report by Qi *et al*., the mixed structures revealed more defects of the nanofibers compared with pure anatase or rutile, which led to the dissociation of more molecules (H_2_O and KCl) and resulted in an increase of carrier concentration, which eventually improved the sensing performance of the nanofibers [146,155].

### 3.8. Zinc-Based Materials for Humidity Sensors

Zinc oxide (ZnO) and TiO_2_ are both very popular semiconductor materials in the field of humidity sensor applications. High chemical and physical stability, good sensitivity as well as fast response and recovery time, and high surface to volume ratio are the main salient features of ZnO-based humidity sensors. However, the higher hydrophobicity of ZnO materials is an inherent drawback, but this can be overcome by using different doping techniques. Different kinds of zinc oxide-based materials for humidity sensors and their synthesis techniques are listed in Table 8. The electrical characteristics (resistance, capacitance, sensitivity, response time, hysteresis, stability) changes with the variation of frequency, temperature and relative humidity of zinc-based sensor materials are explained in the following subsections.

#### 3.8.1. Resistance or Impedance Variation with Relative Humidity

**Figure 44.**
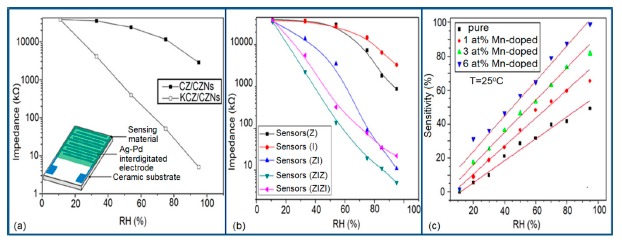
Impedance variation with relative humidity at different concentrations: (**a**) pure and KCl-doped Cu–Zn/CuO–ZnO nanoparticles measured at 1 V, 100 Hz; the inset shows the structure of the humidity sensor applied in our measurement [161]; (**b**) Microhumidity sensors with various film coating forms [164]; (**c**) ZnO nanopowders with different contents of Mn [165].

Like other sensor materials ZnO-based materials have shown decreasing resistance or impedance with increasing RH. The doping or coating materials and their structure or synthesis techniques play important roles in the resistance or impedance *vs.* RH characteristics. A sensor with its substrate, electrodes and ZnO-based sensing material is shown in the inset of Figure 44a. The effect of different concentrations of doping agent (K^+^) in Cu−Zn/CuO−ZnO nanoparticles (CZ/CZNs) on the impedance-RH is depicted in Figure 44a [161]. The KCl-doped Cu−Zn/CuO−ZnO nanoparticles (KCZ/CZNs) show much better linearity of the correlation curve than that of undoped CZ/CZNs on a semi-logarithmic scale. It has been found that the impedance of KCZ/CZNs decreases linearly by about four orders of magnitude as the RH increases from 11% to 95%. Similar behavior was also found in the KCl-doped ZnO nanofiber material [159] and LiCl-doped ZnO nanofibers [160]. In both cases, the doped ZnO nanofibers exhibited greatly improved sensitivity with lower impedance and higher linearity compared to a pure ZnO sample. The impedance-RH behavior of ZnO-based thin films with different coatings or layers for different ZnO−In_2_O_3_ thin film materials is depicted in Figure 44b [164]. Liang *et al.* developed five different films sensors based on fabrication processes such as sputtering of only ZnO (*i.e.*, represented as Z), sputtering of only In_2_O_3_ (*i.e.*, represented as I), sputtering of ZnO + In_2_O_3_ (*i.e.*, represented as ZI), sputtering of ZnO + In_2_O_3_+ ZnO (*i.e.*, represented as ZIZ), and sputtering of ZnO + In_2_O_3_ + ZnO + In_2_O_3_ (*i.e.*, represented as ZIZI). The sensors Z (*i.e.*, ZnO) and I (*i.e.*, In_2_O_3_) show distinct changes in impedance with respect to the humidity only above 54% RH. By depositing both ZnO and In_2_O_3_ films on sensor substrates, the sensor performance was enhanced greatly. The ZI, ZIZ, and ZIZI sensors showed much enhanced signal changes with better linear correlation curves, confirming that the complex film structures directly contribute to the increase of conductivity and the improvement of the linearity. The highest sensing performance was found for ZIZ sensors, which present the best linearity and largest impedance change (see Figure 44b). Addition of Mn in ZnO_2_ enhances its humidity sensing characteristics. From Figure 44c it is observed that increased concentration of Mn increases the sensor sensitivity. The 6 at % Mn-doped ZnO gives the best linearity and highest sensitivity.

#### 3.8.2. Effect of Frequency on Resistance-RH Characteristics

The resistance or impedance of the ZnO-based humidity sensors decreases with increasing RH and it is more pronounced at higher frequency. Furthermore, the change in impedance between the operating frequencies becomes progressively smaller with increasing RH. The probable reason for this result is that at higher frequencies, the adsorbed water cannot be polarized so fast and thus, the dielectric phenomenon does not appear. Most of the ZnO-based sensors, such as flower-like ZnO nanorods [158], KCl-doped ZnO nanofibers [161], LiCl-doped ZnO nanofibers [160], KCZ/CZNs [161], porous ZnAl_2_O_4_ nanorods [167] and ZnO nanotips [169], shows the best linearity in the impedance *vs.* RH curve at nearly 100 Hz.

**Figure 45.**
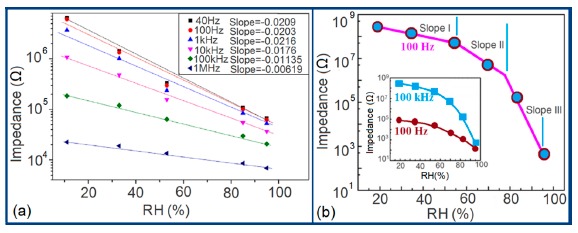
(**a**) Linear variation in impedance variation with relative humidity at different frequencies for ZnAl_2_O_4_ nanorods [167]: (**b**) The different impedance variation *vs.* RH curve slopes at a constant frequency or different frequencies (inset) for ZnO nanotips.

The dependence of impedance on RH for the porous ZnAl_2_O_4_ nanorod-based sensor measured at various frequencies is shown in Figure 45a [167]. The impedance of this sensor decreases with increasing RH at all measuring frequencies. Especially, at low frequency, e.g., at 40 Hz, it changes from 7 × 10^3^ to 70 kΩ as RH varies from 11% to 95%, implying a relatively high sensitivity. Although the impedance was nonlinear with increasing RH, the impedance followed a logarithmic increase with increasing RH and exhibited a good linear log(impedance) relationship to RH in the 11%–95% RH range, as shown by Cheng *et al.* Moreover, the slope of the linear fit curves decreased from −0.02309 to −0.00619 with the increase of measurement frequency from 40 Hz to 1 MHz, clearly indicating a decrease in sensitivity. On the other hand, ZnO nanotip sensors show an impedance change by six orders of magnitude at 100 Hz compared to operating at a frequency of 100 kHz measured at 1 V, as shown in Figure 45b [169]. From the impedance-RH curve of the ZnO nanotips sensor, it can be clearly seen that the impedance of the film decreases remarkably at higher frequency in the low RH range, and the impedance difference between the two working frequencies becomes progressively smaller with increasing RH.

#### 3.8.3. Effect of Frequency on Capacitance-RH Characteristics

The capacitance variation is found to be more at the low frequency range and its effect becomes slow at higher frequency range. This happens due to the sluggish change in electrical field direction at low frequency which strongly affects the space–charge polarization of adsorbed water. A higher RH means more the water molecules are adsorbed and the polarization is stronger, and thus a larger dielectric constant or capacitance value is obtained. However, at a high frequency the polarization of the water cannot catch up with the faster change in electric field direction, and hence the dielectric constant or capacitance value becomes smaller and independent of RH.

**Figure 46.**
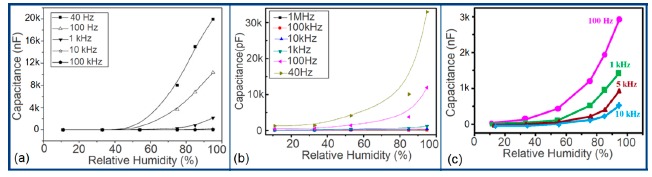
Capacitance variation with relative humidity at different frequencies for different sensor materials: (**a**) Flower-like ZnO nanorods [158]; (**b**) Porous ZnAl_2_O_4_ nanorods [167]; (**c**) ZnO/Si-NPA.

The properties of capacitance *vs.* frequency at different RH values for flower-like ZnO nanorod [158], porous ZnAl_2_O_4_ nanorod [167] and ZnO/Si-NPA [170] sensor materials are depicted in Figure 46a–c, respectively. The flower-like ZnO nanorod sensor shows a large capacitance change at lower frequency (40 and 100 Hz) in the range of 54%–95% RH. The porous ZnAl_2_O_4_ nanorod sensor shows a large change of capacitance at a frequency lower than 100 Hz in the 55%–95% RH range. The ZnO/Si-NPA sensor shows the best capacitance-RH characteristics at 1 kHz in the 55%–95% RH range.

#### 3.8.4. Response and Recovery Time Analysis

**Figure 47.**
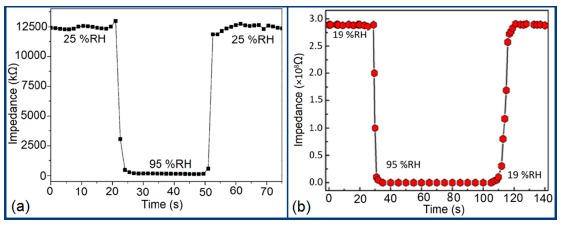
Response and recovery time characteristics of humidity sensors for different materials: (**a**) KCl-doped ZnO nanofiber [159]; (**b**) ZnO nanotip [169].

The response and recovery time of the different ZnO-based humidity sensors are listed in Table 8. The response and recovery characteristics of KCl-doped ZnO nanofiber- [159] and ZnO nanotip [169]-based sensors are depicted in Figure 47a,b, respectively. It has been noticed that the response and recovery times of the KCl-doped ZnO nanofiber sensor is remarkably low compared to any other humidity sensor material.

#### 3.8.5. Hysteresis Characteristics

**Figure 48.**
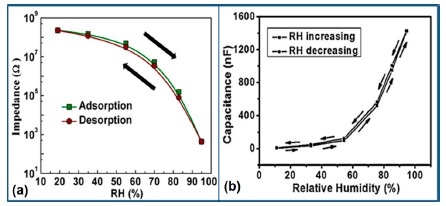
A comparison of the hysteresis loss behavior for different humidity sensor materials: (**a**) impedance-RH (for ZnO nanotip) [169]; (**b**) capacitance-RH (for ZnO/Si-NPA) [170].

The hysteresis of ZnO-based humidity sensors are listed in Table 8. Most of the ZnO-based humidity sensors show excellent hysteresis loss in their impedance or capacitance-RH plots (near to 2%). A comparison of the hysteresis loss behavior between impedance-RH (for ZnO nanotip) and capacitance-RH (for ZnO/Si-NPA) characteristics is depicted in Figure 48a,b, respectively [169,170].

#### 3.8.6. Stability Analysis

**Figure 49.**
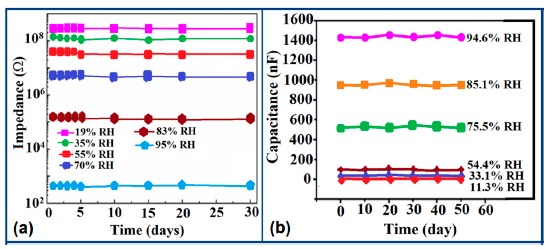
Schematic representation of stability behaviour in capacitance-time plots at different RH for different doped sensor materials: (**a**) ZnO nanotip; (**b**) ZnO/Si-NPA.

In the low humidity range, there was almost no change observed even up to 60 days in the impedance or capacitance values, which directly confirms the good stability of ZnO-based humidity sensors. A slight fluctuation is observed in the higher RH range above 50% RH. The impedance stability of the ZnO nanotip-based humidity sensor in a wide range of RH values was observed up to 30 days at 100 Hz as depicted in Figure 49a [169], indicating good stability and durability of the sensor.

A good long term stability was also observed for a ZnO/Si-NPA cauliflower humidity sensor, which showed insignificant change in capacitance values up to 50 days exposure to ambient air at temperatures in between 19 °C and 30 °C (see Figure 49b) [170].

### 3.9. Zirconia-Based Materials for Humidity Sensors

Many kinds of materials like ceramics, organic polymers, semiconductors, and solid electrolytes have been studied for humidity analysis. However, recently zirconia (ZrO_2_) has being selected for advanced humidity sensors due to its unique properties, such as high thermally and excellent chemically stability, high toughness, high strength, and good catalytic activities, which make it more attractive compared to other materials in wide range of applications. Very little work has been reported on ZrO_2_-based humidity sensors so far. Wang *et al.* [171] designed a ZrO_2_ thick film humidity sensor, which was made of ZrO_2_ nanoparticles with an average grain size 20 nm, was used to deposit it as a thick film on a silicon substrate. Y^3+^-doped and Mg^2+^-doped zirconia thick film humidity sensors have also been investigated by Su *et al.* [172]. The changes in electrical characteristics (resistance, capacitance, sensitivity, response time, hysteresis, stability) with the variation of frequency, temperature and relative humidity of zirconia-based sensor materials are explained in the following subsections.

#### 3.9.1. Effect of Temperature on Resistance-RH Characteristics

**Figure 50.**
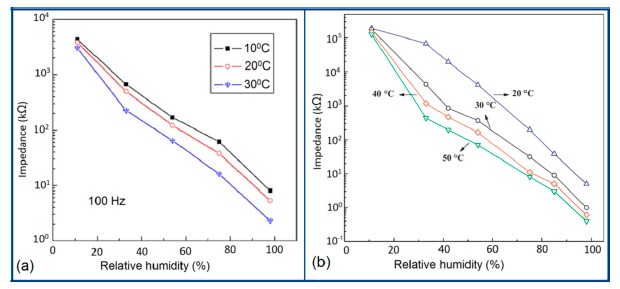
Impedance variation with relative humidity at different temperatures: (**a**) for ZrO_2_ [171]; (**b**) for Y^3+^-doped and Mg^2+^-doped ZrO_2_ humidity sensors [172].

The resistance or impedance of ZrO_2_-based sensor materials decreases with increasing RH and temperature. The trend is similar to that of other sensor materials. It has also been found that the sensitivity of the doped ZrO_2_ thick film is better than that of bare ZrO_2_ humidity sensors, but the slope of the curves hardly changes with temperature, indicating that the sensitivity of the ZrO_2_ sensor does not change with temperature in this temperature range from 10 °C to 30 °C (see Figure 50a) [171]. On the other hand, the impedances of a Y^3+^-doped and Mg^2+^-doped ZrO_2_ sensors are larger than the those of an undoped one and at a particular temperature the impedance of the doped sensors varied almost five orders of magnitude from 10^8^ to 10^3^ Ω when the RH increased from 11% to 98% RH (see Figure 50b) [172].

#### 3.9.2. Effect of Frequency on Resistance-RH Characteristics

**Figure 51.**
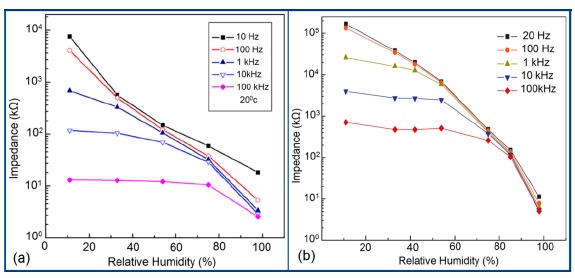
Impedance variation with relative humidity at different frequencies for different doped sensor materials: (**a**) undoped ZrO_2_ [171]; (**b**) Y^3+^-doped ZrO_2_ [172] humidity sensors.

Like the other materials, the impedance decreases as the frequency increases and the impedance became independent of the humidity with increasing frequency. It has been found that the best linearity of the impedance–RH characteristic appears at frequencies between 100 Hz and 1 kHz for ZrO_2_ sensors [171], however, the best linearity is observed in the 20–100 Hz range for yttrium (Y^3+^)-doped ZrO_2_ humidity sensors [172], as depicted in Figure 51a,b, respectively.

#### 3.9.3. Response and Recovery Time Analysis

The response and recovery times of ZrO_2_-based sensors change strongly with doping agent. For example, the response time of a Y^3+^-doped ZrO_2_ humidity sensor is 30 s and for a Mg^2+^-doped sensor it is 5 s; and the recovery time for both the Y^3+^ and Mg^2+^-doped ZrO_2_ sensor is 5 s [171]. The response and recovery time of a pure ZrO_2_ humidity sensor are 130 s and 60 s, respectively [172]. Therefore, comparatively the Mg^2+^-doped ZrO_2_ sensor has the smallest response and recovery time, and can be considered as the best compared to the pure ZrO_2_ or Y^3+^-doped ZrO_2_ sensors.

#### 3.9.4. Hysteresis Characteristics Analysis

The humidity hysteresis of the pure ZrO_2_ sensor is about 8% RH [171], while on the other hand it is 3% RH and 4% RH for Y^3+^- and Mg^2+^-doped ZrO_2_ sensors, respectively [172]. Therefore, considering the overall properties of the all ZrO_2_-based ceramic sensors, for less hysteresis value of a Y^3+^-doped sensor can be selected as the most suitable material for humidity measuring devices.

### 3.10. Sodium-Doped Crystalline Materials for Humidity Sensors

Oxide ceramic materials have mostly been used in humidity sensor applications. Sodium oxide-based sensors also are being tried in humidity sensing devices due to its attractive thermal, physical and chemical stability like that of other ceramic materials. However, the main drawback of normal ceramics-based humidity sensors is their insufficient sensitivity over a wide humidity range, as well as lack of reversibility and drift in base resistance with time due to chemisorption of water molecules. Therefore, to overcome this problem many researchers are trying to use sodium-based materials to promote the sensitivity. Among them Na- and K-montmorillonite [173] sensors are the most suitable sensors for practical application. Zhang *et al.* [174] developed a novel humidity sensor based on nanocrystalline NaTaO_3_. The changes in electrical characteristics (resistance, capacitance, sensitivity, response time, hysteresis, stability) with the variation of frequency, temperature and relative humidity of sodium-based sensor materials are explained in the following subsections.

#### 3.10.1. Effect of Temperature on Resistance-RH Characteristics

**Figure 52.**
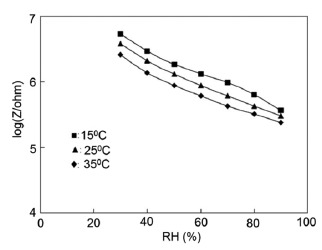
Impedance *vs.* relative humidity plots of a montmorillonite sensor at various temperatures, measured at 1 V and 1 kHz [173].

Su *et al.* [173] observed that the impedance of the Na-montmorillonite sensor depends on the ambient temperature (see Figure 52). When the temperature increased, the RH characteristic curve shifted to the lower impedance side which may be because the thermal energy is applied to activate the mobile charge carriers within a stable water layer. The average temperature coefficient between 15 °C and 25 °C was −0.23% RH/°C in the humidity range of 30%–90% RH.

#### 3.10.2. Effect of Frequency on Resistance-RH Characteristics

In both pure Na-montmorillonite- [173] and NaTaO_3_-based humidity sensors [174], the impedance decreases with the increase of relative humidity value in the range of 33%–95% RH. It is observed that the NaTaO_3_-based humidity sensor has a better linearity than the pure Na-montmorillonite one.

**Figure 53.**
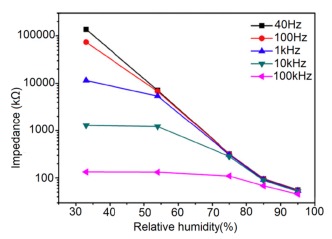
The relationship between impedance and RH of NaTaO_3_ was measured at various frequencies [174].

In the Na-montmorillonite sensor the best linearity was observed at 1 kHz, but in the NaTaO_3_-based sensor the best linearity is observed at 100 Hz (see Figure 53), so the NaTaO_3_-based sensor has more sensitivity and better linearity.

#### 3.10.3. Response and Recovery Time Analysis

The response and recovery time of the pure Na-montmorillonite sensor are 40 and 120 s, respectively [173], but for NaTaO_3_-based sensor the response time is 3 s and the recovery time is 32 s [174], confirming that the NaTaO_3_-based sensor has excellent response and recovery time.

#### 3.10.4. Hysteresis Characteristics Analysis

**Figure 54.**
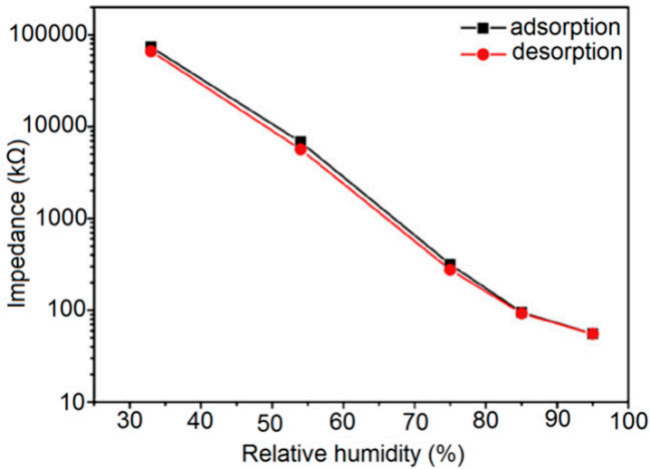
Humidity hysteresis characteristic of a NaTaO_3_ sensor measured at 100 Hz [174].

The hysteresis values of a NaTaO_3_-based sensor and Na-montmorillonite sensor are 1% RH [174] and 3.32% RH [173], respectively. For a good humidity sensor, the hysteresis value must be as small as possible, so this confirms that a NaTaO_3_-based sensor has a better hysteresis response (see Figure 54). The addition of dopant with Na material enhances its characteristics.

## 4. Applications and Scopes of Humidity Sensors

After analyzing the different electrical characteristics of different doping concentrations of different materials, it is very important to study the wide range of applications where humidity sensors are used. Humidity sensors have several applications and scopes such as medical, agricultural, mineral, fuel, aerospace, high-energy physics, and food storage of humidity sensors have been iscussed [175–183]. The typical humidity sensors, including flexible micro-humidity sensors [15,27,126,127,129,130,135,136,184–190], with different humidity or moisture sensing mechanisms, operating temperatures and different humidity ranges have been used in wide range of applications [184–190].

### 4.1. Medical Applications

The significance of environmental factors on the health of human populations depends significantly on the different environmental factors. However, humidity is an important parameter for the detection of diseases. Several researchers have focused on the different applications of humidity sensors in different fields of medical science [175–183].

To monitor respiratory disorders and failures, e.g., hypopnoea and apnoea disease, Kang *et al.* [178] developed a thin film optical humidity sensor using the novel electrostatic self-assembly (ESA) technique.

Sweating generally creates an uncomfortable environment for prosthetic device users. Generally, the use of prosthetic devices for a long time in young or active patients creates a huge sweating problem at the used portion of their body. Sweating rate measurement is also a challenge for researchers. It can be measured by using different humidity sensors to avoid the discomfort of the prosthetic users.

Advanced telehomecare (THC) systems use humidity sensors to monitor serious changes in chronic conditions as well as other health risks including floods, fires and gas leaks. A team led by Haick at the Israel Institute of Technology (Technion) made a humidity sensor breathing tests to detect lung cancer which contains multiple gold nanoparticle-based chemiresistors that can detect chemicals through changes in electrical resistance. The approach for breath-volatile organic compound (VOC) collection and preconcentration by applying needle traps was developed and optimized by Filipiak *et al.* [179]. Here humidity sensors are used for moisture measurement in exhaled breath. Ammonia (NH_3_) is an important biomarker which has a significant role in the human body. Ammonia is present in all body fluids, mainly as ammonium ion (NH_4_^+^) but also in the form of NH_3_. A high concentration of NH_3_ is toxic to the human body. During the last decades, considerable effort has been put into detecting gaseous exhaled breath ammonia (eNH_3_) as a surrogate for blood or urine detection. Schmidt *et al.* [180] developed a humidity sensor-based experimental setup for the detection of ammonia in breath and emitted from the skin.

Breath monitoring is essential during certain imaging and surgical procedures where the patient needs to be sedated or anesthetized [175]. Humidity balance is very important for an anesthetized patient. Consider breathing air controllers in a medical environment (anesthesia), where only small variations in relative humidity content of the breathing air would result in nausea of the patient or even unfavorably impact the anesthetic result. Anesthesia machines and respirators generally require fully humidified air, which enters the patient's lungs. The HIH-3610 series humidity sensors are used to measure the relative humidity near the point of condensation, but the sensor output is not accurate, so a suitable humidity sensor is very important to measure the humidity level.

To study the progression of a diagnosed illness or to evaluate the health of a person it is very important to monitor the breath. It is not at all wise to use electronic breathing sensors when patients are in a magnetic resonance imaging (MRI) system, or during any oncological treatment, which may create hazards for the patient [181]. A simple photonic crystal fiber (PCF) interferometric breathing sensor was developed by Favero *et al.* [181] and an agarose-based optical fiber interferometeric humidity sensor was demonstrated by Mathew *et al.* [182].

The most familiar and common form of communication is voice communication. Due to some hereditary reasons or acquired impediments or due to other reasons like an accident, there are many people who suffer from speech/hearing difficulties. A humidity sensor-based language recognition system that focuses on the moisture included in devoiced breaths as a method for communication support in persons with speaking difficulties was developed by Morisawa *et al.* [183].

### 4.2. Tissue Engineering

Humidity controlled incubators are one of the essential pieces of equipment in tissue engineering applications. Normally 50%–60% RH is maintained for growing animal cells in the incubator.

### 4.3. Food Process and Storage Applications

The shelf life of fruits and vegetables may change with humidity. Excessive water loss from fruits during storage or transportation often reduces its shelf-life. Depending on the type of fruits and the storage methods, the environment can be infected with different bacteria [191]. Therefore, controlling the humidity during the transportation and storage of fruits is a potential use in the agricultural sector and thus proper humidity sensors that measure the availability of water to micro-organisms are used to give an indication of the biological activity, or potential activity, of the product at high RH (above 75% RH) [192].

### 4.4. Agricultural Applications

Analysis of the root distribution of plant and its water absorbing ability is an important subject of considerable interest in ecology and agriculture. It gives a better understanding of the behaviour of different crops under sub-optimal environments, so that the improvement of the quality of modelling of root water uptake in different hydrological environment is possible [193]. The effect of soil moisture on corn root growth was investigated by Mackay *et al.* [194] and it was observed that, due to the increase of soil moisture, the total plant weight increased by 13%–43% and the corn root length increased from 41% to 52% in 28 days.

### 4.5. Structural Health Monitoring (SHM) Applications

The most widely used areas of humidity and moisture sensors are in structural health monitoring (SHM) [195–197] applications. Over the past few decades, the deterioration of civil infrastructure, such as buildings, bridges and roadways have demonstrated the need for high-performance sensing systems in order to protect from corrosion by passivation of steel rebar surfaces, which are embedded in the concrete, due to the high alkalinity of the concrete.

### 4.6. Ecological Applications

To reduce erosion in mountain areas humidity sensors is used by different regulated methods such as check dams, often made of concrete or wood-logs, which decrease the water speed during storm events, allowing sediment to settle [198].

### 4.7. Mineral Processing Applications

The study of moisture content in soil has also been a subject of considerable interest in mineral processing plants. Manual gravimetric drying moisture determination methods are still currently employed by most mineral processing plants, but they fails to provide timely and accurate information [199]. An efficient method of on-line moisture content monitoring such as a fibre-optic moisture sensor would be an ideal tool for such mineral processing applications.

### 4.8. Fuel Applications

In many ways, biomass is a new source of power. Biomass is the energy which is contained inside plants and animals. The variation of moisture contents affects its energy-producing ability. The moisture content in biomass fuel is therefore an important parameter, which often fluctuates [200]. By using direct measurement on the entering fuel or by measuring the moisture and oxygen contents of the flue gases, the fuel moisture content in a furnace can be determined. Environmental pollution is a big challenge nowadays. Most researchers are now emphasizing green energy development. Fuels that don't produce carbon dioxide are one possible solution. Kuo *et al.* [188,189] developed a non-invasive flexible humidity and temperature microsensor and an *in situ* wireless sensing system for a proton exchange membrane fuel cell (PEMFC) application.

### 4.9. Aerospace Applications

Recently, humidity sensors have been used to perform *in situ* measurements in space, most spectacularly of the near-surface atmospheric water content on Mars [201–203]. The water content of soils significantly influences their chemical and physical properties and is also needed for biological processes to proceed.

### 4.10. High-Energy Physics Applications

The Conseil Européen pour la Recherche Nucléaire (European Council for Nuclear Research or CERN) [204] is the world's leading laboratory for particle physics and is one of the world's largest and outstanding centers for scientific research. A fiber optic humidity sensor for high-energy physics applications was developed at CERN by Consales *et al.* [67]. This work was devoted to a feasibility analysis for the development of novel fiber optic humidity sensors to be applied in high-energy physics (HEP) applications.

### 4.11. Electronics Industry

Humidity controlled dark rooms are used for many electronics materials or electronic devices during manufacturing. Power circuit boards (PCBs) are manufactured in controlled humidity dark rooms. Nowadays for low power consumptions in most of the industrial application, radio frequency identification (RFID) tags [190] based flexible capacitive humidity sensors are used.

## 5. Conclusions

Humidity sensors for measuring relative humidity based on different types of materials, including carbon, vanadium, iron, silicon, polymer, tin, titanium, zinc, zirconia, and sodium have been reviewed extensively. The electrical properties of humidity sensors such as resistance and capacitance, the effects of temperature, frequency, and relative humidity, sensitivity, response time, hysteresis and stability have been compared in detail for various materials. For different sensor materials, the electrical properties are changed significantly with the doping concentration of different materials, film thickness of the substrate and the resulting morphological changes. The basic principle of six different types of humidity sensors and their typical advantages as well as disadvantages are discussed in the miniaturization section. Based on our overall review, the general electrical responses such as resistance, capacitance, hysteresis, response and recovery times, stability and their best characteristic features for different materials with respect to relative humidity, frequency and temperature are listed in Table 9. From the above analysis it has been observed that the different doping materials for humidity sensors like carbon, vanadium, iron, silicon, polymer, tin, titanium, zinc, zirconia and sodium show their best linearity at the optimized frequencies of 1 kHz, 100 Hz, 100 Hz, 100 Hz, 1 kHz, 100 Hz, 100 Hz, 100 Hz, 100 Hz and 1 kHz, respectively. Hysteresis is one of the most important parameters for all sensors. For practical applications a sensor must have minimum hysteresis value. In this review article the best hysteresis value for different humidity sensor doping materials like carbon, vanadium, iron, silicon, polymer, tin, titanium, zinc, zirconia, and sodium were 3.57%, <3%, 4%, 1.99%, 1%, 3%, 3%, 1.9%, 3% and 1%, respectively. The hysteresis for vanadium oxide-based sensors was also lower compared to carbon-based materials. The response and recovery times are also an important parameter for all humidity sensors. The response and recovery times of different doped humidity sensors have been critically reviewed. The best response times for carbon, vanadium, iron, silicon, polymer, tin, titanium, zinc, zirconia and sodium-doped sensor materials were 16, 13, 32, 10, <2, 5, 3, 2, 5 and 3 s, respectively, and best recovery time of these sensor materials was 8, 5, 36, 15, 20, 6, 3, 1, 5 and 32 s, respectively. The selection of sensor material based on hysteresis, response time, recovery time and stability is listed in Table 10. In ordered to select a best humidity sensors based on different materials, according to the F-value and T-test by statistical analyses, we grade three categories such as good (*i.e.*, >3% for hysteresis, >19 s for response and recovery times), very good (*i.e.*, 2%–3% for hysteresis, 6–19 s for response and recovery times) and excellent (*i.e.*, <3% for hysteresis, <6 s for response and recovery times). Since stability is the change in resistance or capacitance curves with respect to humidity over a long time, according to observation, it is directly graded as good, very good and excellent according to our flatness for a particular humidity sensor material.

Although response and hysteresis loss are a little less in polymeric sensors compared to zinc oxide-based humidity sensors, the recovery time is significantly larger in polymeric sensors than that of zinc oxide-based sensor materials. Based on our review, it has been found that the zinc oxide-based sensor material is the best for humidity sensor design due to its extremely low hysteresis loss, minimum response and recovery times, and excellent stability. Humidity sensors have a tremendous range of applications in different important fields such as biomedicine, SHM, food processing and storage, medicine, ecology, agriculture, mineral processing, fuel quality control, aerospace, the electronics industry and high-energy physics applications. The humidity conditions, including temperature and % RH, are also very important for different manufacturing or processing sectors as well as their specific areas. Therefore, the development of more integrated humidity sensors for multi-field applications would be highly fascinating topic for the new generation of humidity sensing devices. The review has provided examples that represent the diversity of applications and the demand for RH or moisture sensors in industry today. Therefore, it can be expected that different advanced humidity sensors made of hybrid or doped materials using the latest nanotechnology will be able to monitor a wide range of application sectors.

## Figures and Tables

**Table 1.** Chronological developments of different optical fiber-based humidity sensors and their important results using different principles and materials.

Year	Type	Material	Response Time	Range (% RH)	Ref.
1985	Evanescent wave	CoCl_2_ doped gelatin film	<1 min	50–80	[31]
1988	Evanescent wave	Porous SiO_2_ optical fibre cladding	-	25–95	[32]
1988	Direct spectroscopic	Etched borosilicate opticalfibre segment doped with CoCl_2_	<5 min	20–50	[33]
1989	Interferometric	SiO2–TiO_2_–SiO_2_ cavity	1 min	0–80	[34]
1991	Interferometric	Nafion	<1 min	-	[35]
1995	Evanescent wave	CoCl_2_ doped gelatin film	1 s	20–80	[36]
1995	Direct spectroscopic	Aluminium/morin metalion–organic complex doped PVP membrane	-	0–80	[37]
1996	Evanescent wave	a surface plasmon waveguide with a thin layer of Nafionfluoropolyme	-	20–50	[38]
1997	Direct spectroscopic	Crystal violet doped Nafion film	-	40–82	[39]
1998	Direct spectroscopic	Rhodamine B doped HPC film	∼2 min	0–95	[40]
1999	Interferometric	SiO_2_–[Au:PDDA + /PSS-]-air cavity using ISAM technique	1.5 s	11–100	[41]
2000	Evanescent wave	Agarose gel	<1 min	30–80	[42]
2001	Interferometric	SiO_2_–[PDDA + /PS-119]-air cavity using ISAM technique	3 s	0–97	
2002	In-fiber grating	Polyimide	-	10–90	[43]
2002	Evanescent wave	CoCl_2_ doped PVA film	-	S: >78 U: 3–90	[44]
2003	Evanescent wave	HEC/PVDF film	<5 s	20–80	[45]
2004	Direct spectroscopic	Porous sol-gel fibre segment doped with CoCl_2_	-	2–10	[46]
2004	Evanescent wave	Porous sol-gel cladding	<1 min	3–90	[47]
2005	In-fibre grating	CoCl_2_ doped PEO film	<1 s	I: 70–80 W: 40–80	[48]
2006	Direct spectroscopic	Ruthenium-based complex doped PTFE membrane	∼2 min	4–100	[49]
2006	Evanescent wave	PDDA/Poly R-478 nanostructured sensing overlay using ISAM technique	-	75–100	[50]
2008	Interferometric	PVA	-	33–97	[51]
2008	In-fiber grating (FBG)	PI	∼25 min	22–97	[52]
2008	Evanescent wave	Gelatin	<0.5 s	9–94	[53]
2008	In-fiber grating (LPG)	PVA	<1 min	33–97	[54]
2008	Absorption	SiO_2_nano-particles	<1 s	75–100	[55]
2009	In-fiber grating (FBG)	PVA	<2 s	20–98	[56]
2009	In-fiber grating (LPG)	Poly(ethylene oxide)/CoCl_2_	<10 s	50–95	[57]
2009	Evanescent wave	ZnO	30 s	5–90	[58]
2009	Grating + interferometric	Hydrogel	-	60–100	[59]
2009	Absorption	ITO	-	20–80	[60]
2010	In-fiber grating (FBG)	PI	-	30–80	[61]
2010	Evanescent wave	Ag-Polyaniline	30 s	5–95	[62]
2010	Absorption	Xerogel	10 s–2 min	10–70	[29]
2011	In-fiber grating (FBG)	PI	-	0–75	[63]
2011	In-fiber grating (LPG)	SiO_2_nano-sphere film	<1 s	20–80	[64]
2011	Evanescent wave	PVA	-	50–89	[65]
2011	Absorption	PVA/SiO_2_/CoCl_2_	<2 min	25–65	[66]
2011	Interferometric	Tin dioxide	-	2–40	[67]
2012	Absorption	Au-NP/boehmite	<20 s	-	[67]
2012	Interferometric	PVA	<6 s	20–80	[68]
2012	Evanescent wave	TiO_2_	<0.5 s	24–95	[69]
2013	Evanescent wave	PAA electrospunnanowires	<0.5 s	30–95	[70]
2013	Grating + interferometric	PI	-	20–80	[71]

**Table 2.** Most important materials and methods used for silicon based humidity sensors.

Form of Silicon	Materials Used	Methods	Ref.
Nanoparticles	Silica aerogel	Sol-gel	[110]
Mesoporous	Li-SBA-15 (0.1 Li^+^)	Sol-gel	[111]
Mesoporous	SBA-16/Li^+^	Chemical synthesis	[112]
Mesoporous	K_2_CO_3_-SBA-15	Thermal dispersion	[113]
Mesoporous	K-SBA-15	Chemical synthesis	[114]
Mesoporous	ZnO–SiO_2_	One-pot sol-gel	[115]
Nanoporous pillar array	nw-SiC/Si-NPA	Chemical vapor deposition	[116]
Nanoporous pillar array	Zn2SiO4/Si-NPA	Chemical vapor deposition	[117]
Mesoporous	Li-A-SBA-15 (0.15 Li^+^)	Chemical synthesis	[118]
Mesoporous	Fe-SBA-15	Chemical synthesis	[119]
Mesoporous	Li-MCM-41	Chemical synthesis	[120]

**Table 3.** Response time, recovery time, and hysteresis of different silicon based humidity sensor materials.

Materials	Condition or Doping Concentration	Response Time (s)	Recovery Time (s)	Hysteresis (%RH)	Ref.
Silica aerogel	coating viscosity of 15 cp	41	55	3.3	[110]
Li-SBA-15 (0.1 Li^+^)	weight ratio of LiCl to SBA-15 = 0.10	21	51	6	[111]
SBA-16/Li^+^	10 wt % Li	25	120	4	[112]
K_2_CO_3_-SBA-15	weight ratio of K_2_CO_3_ to SBA-15 = 0.8	15	50	-	[113]
K-SBA-15	weight ratio of KCl to SBA-15 = 0.5	10	<25	3	[114]
ZnO–SiO_2_	molar ratio of ZnO to SiO_2_ = 1	50	100	2	[115]
nw-SiC/Si-NPA	-	105	85	4.5	[116]
Zn_2_SiO_4_/Si-NPA	-	25	15	1.99	[117]
Li-SBA-15 (0.15 Li^+^)	weight ratio of LiCl to SBA-15 = 0.15	60	180	3	[118]
Fe-SBA-15	weight ratio of Fe(NO_3_)_3_ to SBA-15 = 0.5	20	50	-	[119]
Li-MCM-41	2 wt % Li	100	150	-	[120]
MgO-SBA-15	molar ratio of MgO to SBA-15 = 1	10	20	2	[124]

**Table 4.** Chronological development of different types of polymeric humidity sensors and their manufacture procedures.

Sensor Material	Method	Type	Important Property	Ref.
Silicone-containing polyelectrolyte (Si-PE) copolymer crosslinked with dibromobutane (DBB):	Radical copolymerization	Resistive	Better humidity sensitivity	[125]
Si-PE is a mixture of 2-(methacryloyloxy)-ethyl]dimethyl butyl ammonium bromide (DMAEM-BuBr)				
γ-methacryloxypropyltrimethoxysilane (MPTS) and azodiisobutyronitrile (AIBN)				

Quaternized 3-aminopropyltriethoxysilane (APTS) electrolyte with n-butyl bromide (BB) filmed on gold (Au) coated ceramic substrate	Electrolyte was made by hydrolysis using a simple one-pot method; Filmed was made by automatic dip-coating	Resistive	Highly water-resistive	[128]

Pure polypyrrole (PPy) and TiO_2_ nanoparticles/polypyrrole (TiO_2_NPs/PPy) composite thin films on an alumina substrate	*In situ* photo polymerization	Resistive	Flexible	[129]

TiO_2_ nanoparticles/polypyrrole (TiO_2_NPs/PPy) and TiO_2_ nanoparticles/polypyrrole/poly-[3-(methacrylamino)propyl] trimethyl ammonium chloride (TiO_2_NPs/PPy/PMAPTAC) com-posite thin films on a polyester (PET) substrate	Comb-like structure using *in situ* photopolymerization by ultraviolet (UV) light	Resistive	Flexible	[130]

2-(Dimethylamino) ethyl methacrylate (DMAEMA) polyelectrolyte	Quaternized with n-butyl bromide(BB) followed by copolymerized with1,4-divinylbenzene; cross linked by UV irradiation	Resistive	High sensitivity	[131]

TiO_2_ and polystyrene sulfonic sodium (NaPSS) composite film	Dip-coating	Resistive	Better humidity sensitivity	[132]

Polypyrrole composite	Chemical polymerization at room temperature; followed by quaternization with1,4-bromobutane	Resistive	Suitable for detection of low humidity	[133]

Fe^2+^ doped polypyrrole(PPy), where FeCl_3_·6H_2_O played a role of oxidant	*In situ* polymerization	Resistive	Fast response to humidity change	[134]

1-amine terminated polyamidoamine (PAMAM) dendrimer (G1-NH2)- gold (Au) nanoparticles (G1-NH2-AuNPs) was coated on a polyester (PET) substrate	Coating	Resistive	Flexible	[135]

Anchoring of polyelectrolyte to the Au electrode on plastic substrates; A pair of comb-like Au electrodes on a PET substrate was pretreated with 3-mercaptopropionic acid (MPA) and further reacted with a copolymer of methyl methacrylate (MMA) and [3-(methacrylamino) propyl] trimethyl ammonium chloride (MAPTAC) using *N*-(3-dimethylaminopropyl)-*N*'-ethylcarbodiimide hydrochloride (EDC) as peptide coupling reagent	Peptide chemical protocol	Resistive	Flexible	[136]

**Table 5.** Best response time, recovery time, and hysteresis of different silicon-based humidity sensor materials.

Materials	Response Time (s)	Recovery Time (s)	Hysteresis (% RH)	Ref.
DMAEM–BuBr/DMAEM (2:1 molar ratio) and TDMAEM/MPTS (2:1 molar ratio) copolymer	4	-	-	[125]
Si-PE copolymer crosslinked with DBB in a DMAEM-BuBr/DMAEM molar ratio of 4	-	-	2	[125]
APTS and BB composite	16	25	1	[128]
TiO_2_/polypyrrole composite	40	20	-	[129]
Flexible PMAPTAC/TiO_2_ composite	30	45	2	[130]
DMAEMA and BB	9	32	1	[131]
NaPSS polymer	<2	80	-	[132]
TiO_2_/NaPSS composite	<2	20	Lower compare to NaPSS	[132]
Quaternized polypyrrole composite film	41	120	Wide	[133]
Fe^2+^ doped polypyrrole	20	150	-	[134]
G1-NH_2_-AuNPs film with 50 mg/mL added HAuCl_4_	40	50	2	[135]
Poly-MMA-MAPTAC anchored onto MPA/Au surface with adding 200 mM EDC	15	20	2	[136]

**Table 6.** Different types of tin oxide-based humidity sensors and their synthesis methods.

Sensor Material	Method	Response Time (s)	Recovery Time (s)	Ref.
KCl-doped SnO_2_ nanofibers silver-paladium (Ag–Pd) interdigital electrodes substrate	Electrospinning and calcination; fabricated by screen-printing	5	6	[1]
KNO_3_-doped SnO_2_–LiZnVO_4_	Wet chemical and calcination	<80	100	[137]
SnO_2_–LiZnVO_4_ ceramic	Liquid state	60	100	[138]
La^3+^and K^+^ co-doped Ti_0.9_Sn_0.1_O_2_ thin films on alumina substrates	Sol-gel	-	-	[139]
SnO_2_ nanoparticles	Microwave irradiation	-	-	[140]
KCl-doped nanoporous Ti_0.9_Sn_0.1_O_2_ thin films	Sol-gel	11	14	[141]
ZnSnO_3_ cubic crystallites	Hydrothermal	7	6	[142]

**Table 7.** Different types of titanium oxide-based humidity sensors and their synthesis methods.

Sensor Material	Method	Response Time (s)	Recovery Time (s)	Hysteresis (%)	Ref.
CdTiO_3_ nanofibers	Electrospinning	4	6	≈7	[143]

Bi_0.5_(Na_0.85_K_0.15_)_0.5_Ti_0.97_Zr_0.03_O_3_ (BNKTZ)	Metal–organic decomposition	18	60	4	[144]

Develop a novel humidity sensor based on Na_2_Ti_3_O_7_ nanowires	Hydrothermal	4	5	-	[145]

Pure TiO_2_ and KCl-doped TiO_2_ nano fibers with different crystallographic structures	Electro-spinning and calcination	3	3	-	[146]

Bi_0.5_K_0.5_TiO_3_ (BKT) powder	Chemical solution method	12	25	3	[147]

Pure CaCu_3_Ti_4_O_12_ and Mg-doped CaCu_3_Ti_4_O_12_	Conventional solid state method	Very slow (>882)	Very slow (>234)	-	[148]

Barium titanate (BaTiO_3_) nanofiber	Electro-spinning and calcination	<5	<4	5	[149]

Bi_0.5_Na_0.5_TiO_3_–Bi_0.5_K_0.5_TiO_3_ (BNT–BKT) powder	Metal-organic decomposition	20	60	4	[150]

TiO_2_ and polystyrene sulfonic sodium (NaPSS) composite films on the alumina substrate	Dip-coating	178.1 (for pure TiO_2_)	5.9 (for pure TiO_2_)	-	[151]
		774.9 (for pure ZTNA composites)	19.7 (for pure ZTNA composites)		

ZnO nanorods in core deposited of TiO_2_ in shell (ZnO/TiO_2_) on glass substrates	Hydrothermal growth (reparation of ZnO nanorods) and sol-gel (deposition of anatase TiO_2_ shells)	990.6	35.4	High	[152]

Electrospun TiO_2_nanofiber with metallic electrodes:	Evaporating metal contacts on SiO_2_ layer thermally grown on silicon substrate				[153]
titanium (Ti),		3	5	3	
nickel (Ni),		4	7	5	
and gold (Au)		7	13	15	

**Table 8.** Different types of zinc oxide-based humidity sensors and their synthesis methods.

Sensor Material	Method	Response Time (s)	Recovery Time (s)	Hysteresis (%)	Ref.
Flower-like ZnO nanorods on a ceramic substrate with silver-palladium (Ag–Pd) interdigital electrodes	Screen-printing	5	10	2	[158]
KCl-doped ZnO nanofibers	Electrospinning	2	1	-	[159]
ZnO nanofibers and LiCl-doped ZnO composite fibers on ceramic substrates with carbon interdigital electrodes	Screen-printing	3	6	2	[160]
KCl-doped Cu–Zn/CuO–ZnO (KCZ/CZN) nanoparticles	Wire electrical explosion (WEE)	40	50	4	[161]
High pure ZnO colloidal nanocrystal clusters (CNCs)	Modified hydrolyzation	110	80	-	[162]
Nanocrystalline zinc tungstate (ZnWO_4_) (nanoparticles, nanorods)	Precipitation or hydrothermal	3	50	5.5	[163]
ZnO and In_2_O_3_ thin films on SiO_2_/Si substrates with interdigitated Pt signal electrodes	Radio-frequency sputtering	15	40	4 (for ZIZ)	[164]
Mn-doped ZnO nanopowders	Sol-gel	6	20	4.36 (for 6 at % Mn doping)	[165]
ZnO nanorods (Capacitive type)	Thermal decomposition and Dielectrophoretically manipulation for deposition on the micromachined electrodes pairs	70	20	-	[166]
Porous zinc aluminate (ZnAl_2_O_4_) spinel nanorods	Homogeneous co-precipitation approach followed by a heat treatment at 900 °C	15	30	2	[167]
ZnO colloid spheres (coated on quartz crystal microbalance, QCM)	Self-assembly	38	18	-	[168]
A-plane ZnOnanotip on R-plane sapphire substrate	Metal organic chemical vapor deposition (MOCVD)	3	12	1.9	[169]
ZnO on a template of silicon nanoporous pillar array (Si-NPA), a regular array of ZnO cauliflowers (Capacitive type)	Chemical vapor deposition (CVD)	20	3	4.16	[170]

**Table 9.** The general electrical responses and their best characteristics for different materials.

Parameter	Characteristic	Remark for Best Response
Resistance, Relative humidity (RH)	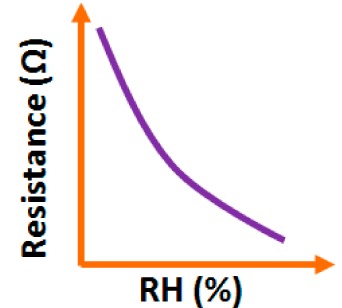	Resistance should decrease with RH

Resistance, Frequency	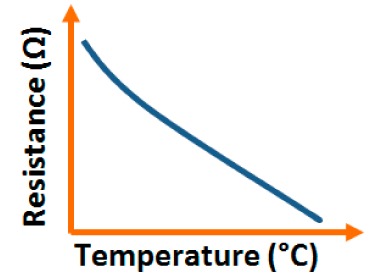	For best response resistance should decreases with frequency

Resistance, Temperature	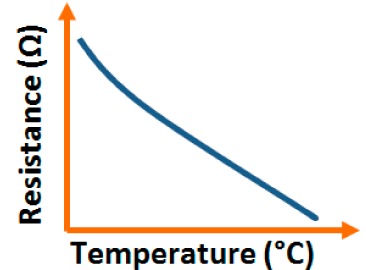	For best response resistance should decreases with temperature

Capacitance, Relative humidity (RH)	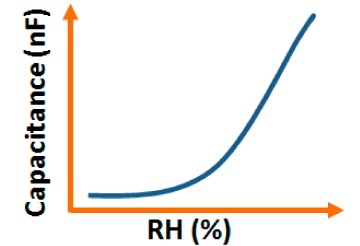	For best response capacitance should increases with RH

Capacitance, Frequency	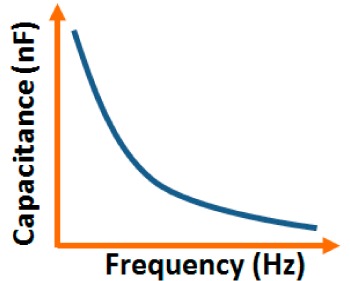	For best response capacitance should decreases with frequency

Hysteresis	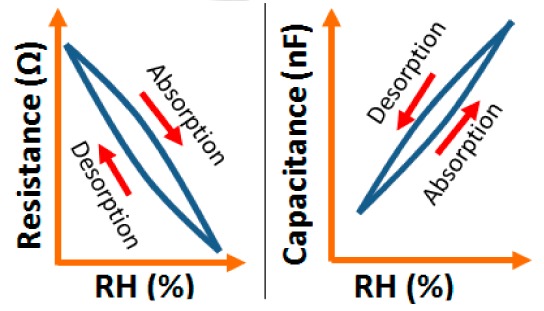	For best response hysteresis loop should be as narrow as possible.

Response and recovery time	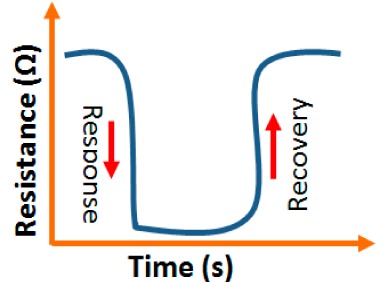	For best response recovery and response time should be as minimum as possible, so that the sensor will give fast response

Repetability	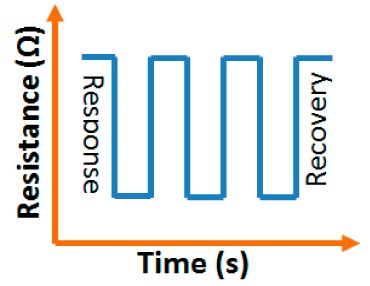	For best response the same response should repeat with time
Stability	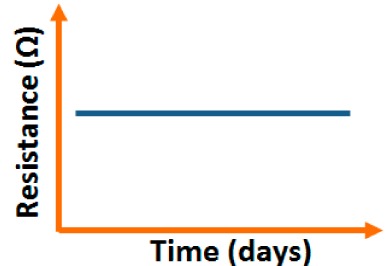	For best stability resistance fluctuation should be minimum with time

**Table 10.** Selection of different sensor materials based on their best electrical response quality: good (*i.e.*, >3% for hysteresis, >19 s for response and recovery times), very good (*i.e.*, 2%–3% for hysteresis, 6–19 s for response and recovery times) and excellent (*i.e.*, <3% for hysteresis, <6 s for response and recovery times).

Sensor Material Based on	Response Time	Recovery Time	Hysteresis	Stability
Carbon	Very good	Very good	Good	Very good
Vanadium	Very good	Excellent	Very good	Excellent
Iron	Good	Good	Good	Very good
Silicon	Very good	Very good	Excellent	Good
Polymer	Excellent	Good	Excellent	Excellent
Tin	Excellent	Excellent	Very good	Good
Titanium	Excellent	Excellent	Very good	Very good
Zinc	Excellent	Excellent	Excellent	Excellent
Zirconia	Excellent	Excellent	Very good	Very good
sodium	Excellent	Good	Excellent	Very good
